# Effect of Mind-Body Skills Training on Quality of Life for Geographically Diverse Adults With Neurofibromatosis: A Fully Remote Randomized Clinical Trial

**DOI:** 10.1001/jamanetworkopen.2023.20599

**Published:** 2023-06-28

**Authors:** Ana-Maria Vranceanu, Heena R. Manglani, Nathaniel R. Choukas, Millan R. Kanaya, Ethan Lester, Emily L. Zale, Scott R. Plotkin, Justin Jordan, Eric Macklin, Jafar Bakhshaie

**Affiliations:** 1 Center for Health Outcomes and Interdisciplinary Research, Department of Psychiatry, Massachusetts General Hospital, Boston; 2 Harvard Medical School, Boston, Massachusetts; 3 Department of Psychology, Harpur College of Arts and Sciences, Binghamton University, Binghamton, New York; 4 Department of Neurology and Cancer Center, Massachusetts General Hospital, Boston; 5 Pappas Center for Neuro-Oncology, Massachusetts General Hospital, Boston; 6 Biostatistics Center, Massachusetts General Hospital, Boston

## Abstract

**Importance:**

Neurofibromatoses (NF; NF1, NF2, and schwannomatosis) are hereditary tumor predisposition syndromes with a risk for poor quality of life (QOL) and no evidence-based treatments.

**Objective:**

To compare a mind-body skills training program, the Relaxation Response Resiliency Program for NF (3RP-NF), with a health education program (Health Enhancement Program for NF; HEP-NF) for improvement of quality of life among adults with NF.

**Design, Setting, and Participants:**

This single-blind, remote randomized clinical trial randomly assigned 228 English-speaking adults with NF from around the world on a 1:1 basis, stratified by NF type, between October 1, 2017, and January 31, 2021, with the last follow-up February 28, 2022.

**Interventions:**

Eight 90-minute group virtual sessions of 3RP-NF or HEP-NF.

**Main Outcomes and Measures:**

Outcomes were collected at baseline, after treatment, and at 6-month and 1-year follow-up. The primary outcomes were physical health and psychological domain scores of the World Health Organization Quality of Life Brief Version (WHOQOL-BREF). Secondary outcomes were the social relationships and environment domain scores of the WHOQOL-BREF. Scores are reported as transformed domain scores (range, 0-100, with higher scores indicating higher QOL). Analysis was performed on an intention-to-treat basis.

**Results:**

Of 371 participants who underwent screening, 228 were randomized (mean [SD] age, 42.7 [14.5] years; 170 women [75%]), and 217 attended 6 or more of 8 sessions and provided posttest data. Participants in both programs improved from baseline to after treatment in primary outcomes of physical health QOL score (3RP-NF, 5.1; 95% CI, 3.2-7.0; *P* < .001; HEP-NF, 6.4; 95% CI, 4.6-8.3; *P* < .001) and psychological QOL score (3RP-NF, 8.5; 95% CI, 6.4-10.7; *P* < .001; HEP-NF, 9.2; 95% CI, 7.1-11.2; *P* < .001). Participants in the 3RP-NF group showed sustained improvements after treatment to 12 months; posttreatment improvements for the HEP-NF group diminished (between-group difference for physical health QOL score, 4.9; 95% CI, 2.1-7.7; *P* = .001; effect size [ES] = 0.3; and psychological QOL score, 3.7; 95% CI, 0.2-7.6; *P* = .06; ES = 0.2). Results were similar for secondary outcomes of social relationships and environmental QOL. There were significant between-group differences from baseline to 12 months in favor of the 3RP-NF for physical health QOL score (3.6; 95% CI, 0.5-6.6; *P* = .02; ES = 0.2), social relationships QOL score (6.9; 95% CI, 1.2-12.7; *P* = .02; ES = 0.3), and environmental QOL score (3.5; 95% CI, 0.4-6.5; *P* = .02; ES = 0.2).

**Conclusions and Relevance:**

In this randomized clinical trial of 3RP-NF vs HEP-NF, benefits from 3RP-NF and HEP-NF were comparable after treatment, but at 12 months from baseline, 3RP-NF was superior to HEP-NF on all primary and secondary outcomes. Results support the implementation of 3RP-NF in routine care.

**Trial Registration:**

ClinicalTrials.gov Identifier: NCT03406208

## Key Points

**Question:**

Does a remote mind-body skills training program improve quality of life more than a health education program for adults with neurofibromatosis?

**Findings:**

In this randomized clinical trial that included 228 adults with neurofibromatosis, both the mind-body and education programs significantly improved physical and psychological quality of life. However, only improvements in the group who received the mind-body program remained significant through 12 months of follow-up.

**Meaning:**

These findings suggest that a mind-body skills training program tailored to patients with neurofibromatosis can have long-term benefits on physical and psychological quality of life.

## Teaser

This remote randomized clinical trial compares a mind-body skills training program with a health education program for improvement of quality of life among adults with neurofibromatosis.

## Introduction

Neurofibromatoses (NF; NF1, NF2, and schwannomatosis) are nervous system disorders unified by risk of nerve sheath tumors^1^ that cause significant morbidity, including disfiguring cutaneous tumors (NF1)^1,2^; complete hearing loss, facial weakness, and poor balance (NF2)^3^; and chronic pain (schwannomatosis).^4^ A change in disease nomenclature is underway that considers NF2 a subtype of schwannomatosis (NF2-related schwannomatosis) based on its genetic properties; we retain the 3 subtypes to stratify randomization in the current trial. Neurofibromatoses have no cure.^5^

Neurofibromatoses share poor quality of life (QOL),^6,7^ depression, anxiety, and stress associated with symptom burden,^8^ difficulties getting appropriate medical care,^9^ and social isolation.^10^ Very few pilot and no fully powered efficacy psychosocial randomized clinical trials (RCTs) of adults with NF have been published.^11,12^

We used a sequential approach to develop and optimize the Relaxation Response Resiliency Program for Neurofibromatosis (3RP-NF), a remote psychosocial program to improve QOL among geographically diverse patients with NF. Using qualitative interviews, an open pilot with exit interviews,^11^ and a pilot RCT,^12^ we developed and refined the program, established its feasibility and acceptability, and showed preliminary and sustained efficacy in improving QOL vs a health education control called the Health Enhancement Program for NF (HEP-NF).^12^

We aimed to establish the efficacy and durability of 3RP-NF vs HEP-NF over 12 months among English-speaking adults with NF worldwide. We hypothesized that participation in 3RP-NF would be associated with improvement in the primary (physical health and psychological QOL) and secondary (social relationships and environmental health QOL) outcomes compared with those participating in the HEP-NF program and that these improvements would persist at 6- and 12-month follow-up.

## Methods

Details of the study design have been previously described.^13^ This study follows the Consolidated Standards of Reporting Trials Extension (CONSORT Extension) reporting guideline. The study was ethically reviewed and approved by the institutional review board at Massachusetts General Hospital. The trial protocol is available in Supplement 1. Participants provided written informed consent, did not receive compensation, and met virtually with study staff to review eligibility and safety protocols.

### Recruitment and Participants

Advertisements were distributed via listservs through the NF registry within the Children’s Tumor Foundation and global NF centers. A total of 993 patients responded to the flyer, 371 were screened, 286 were eligible, and 228 signed informed consent. Inclusion criteria were (1) diagnoses of NF1, NF2, or schwannomatosis; (2) age 18 years or older; (3) ability to provide informed consent; (4) sixth-grade English reading level or higher; (5) self-reported stress and difficulties coping with NF symptoms; and (6) Perceived Stress Scale score of 6 or higher.^14,15^ Exclusion criteria were (1) severe psychopathologic conditions that would interfere with study procedures, (2) change in antidepressants in the past 3 months, (3) participation in cognitive behavioral or relaxation therapy in the past 3 months, (4) significant mental health conditions requiring immediate treatment based on self-report and observation during screening, and (5) unwilling or unable to participate in virtual programs. Participants self-reported their race and ethnicity, which was collected to contribute to statistics on the prevalence of NF. Enrollment occurred October 1, 2017, to January 31, 2021, with the last follow-up February 28, 2022.

### Measures and Data Collection

#### Primary Outcomes

The 2 primary outcomes were the physical health and psychological QOL domain scores of the World Health Organization Quality of Life Brief Version (WHOQOL-BREF),^16,17^ recommended by the Response Evaluation in Neurofibromatosis and Schwannomatosis (REiNS) International Collaboration.^18^ The physical health domain (7 items) assesses activities of daily living, dependence on medical substances or aids, energy and fatigue, mobility, pain and discomfort, sleep and rest, and work capacity. The psychological domain (6 items) assesses enjoyment in life, meaningfulness of life, ability to concentrate, completely accepting bodily appearance, satisfaction with self, and frequency of negative feelings. Scores are reported as transformed domain scores (0-100), with higher scores indicating higher QOL. The WHOQOL-BREF is a reliable, valid, and shorter version of the original WHOQOL-100.^17^ Because an NF-specific minimal clinically significant difference (MCID) does not exist for NF, we cautiously used the MCID value of 6.26 units established for patients with cancer^19^ to depict clinical significance.

#### Secondary Outcomes

Secondary outcomes included social relationships QOL and environment QOL as measured by the respective domains of the WHOQOL-BREF.^16,17^ The social relationships QOL domain (3 items) measures satisfaction with personal relationships, availability of social support, and satisfaction with sexual relationships. The environment QOL domain (8 items) assesses financial resources, physical safety and security, accessibility and quality of health and social care, home environment, opportunities for acquiring skills and information, leisure activities, physical environment, and transportation.

#### Data Collection

Assessments were completed at baseline, after treatment, and at 6- and 12-month follow-up via REDCap.^20^ A research assistant answered any questions. Questionnaires were sent 3 times and followed up with a telephone call, after which unresponsive participants were considered lost to follow-up.

#### Fidelity

We followed National Institutes of Health Behavioral Change Consortium guidelines.^21^ Fidelity of the design was ensured through weekly team meetings to review participants’ progression through the study as well as monthly quality control checks. Fidelity of training was ensured by selecting as interventionists PhD-level or advanced doctoral students in clinical psychology with expertise in mind-body therapy who attended weekly supervision. Treatment delivery was ensured by the completion of an adherence checklist after a session; 15% of all sessions were randomly selected to be reviewed for adherence.

### Intervention and Control Conditions

Eight weekly virtual group sessions in each condition lasted 90 minutes per session. The therapists were 5 PhD-level interventionists (including E.L.) trained and supervised by a senior clinical psychologist (A.-M.V.) with expertise in NF and mind-body medicine. Both study treatments included rehearsal, repetition, and review of previously learned skills. Participants in both conditions received a patient manual designed for a sixth-grade comprehension level to accommodate participants with learning disabilities and other cognitive issues.

#### Intervention

The 3RP-NF has 3 core components: (1) relaxation response–elicitation (relaxation and mindfulness skills), (2) appraisal and coping (adaptive coping skills), and (3) growth enhancement (acceptance, problem-solving, and positive psychology skills) aimed to improve QOL. Each session included a relaxation exercise, a review of previously taught skills, and an introduction of a new skill, and ended with a short relaxation exercise. The final session reviewed all skills, emphasizing continuing skill practice after the end of the program. Additional information on the intervention is presented in Supplement 1 and eAppendices 1, 2, and 3 in Supplement 2.

#### Active Control

The HEP-NF included educational information on NF from the Children’s Tumor Foundation and the Centers for Disease Control and Prevention websites. Modules provided education on NF-related stress and symptoms, sleep, nutrition, exercise, communication, and health care management. The last session included information on ways to maintain positive health changes made during the program.

### Randomization

A randomization schedule with a 1:1 ratio of assignment to 3RP-NF and HEP-NF, stratified by NF type using a permuted-block design, was constructed by the unblinded biostatistician (E.M.) and managed with REDCap. A clinical research coordinator randomized up to 16 participants as a cohort and subsequently scheduled a treatment session time. Because not all participants could join the same session time, group sizes were uneven (median, 8 [range, 3-8]). Participants were told that they would participate in 1 of 2 stress and symptom management programs (program 1, intervention; program 2, control) to maintain blinding and scientific rigor.

### Statistical Analysis

The effective SDs for the change in physical health and psychological QOL scores from preliminary data (baseline to after treatment, 14.7 and 10.4 units, respectively^12^; and after treatment to the 6-month follow-up, 11.4 to 10.0 units, respectively) estimated that 224 participants provided 80% power for primary outcomes of physical health QOL and 96% power for psychological QOL. This assumed an MCID of 6.25 units, allowing up to 5% loss to follow-up after treatment and testing each of the primary outcomes at *P* < .025 (2-tailed). Expecting up to 20% of participants to drop out before 6 months, the trial had 99% power to declare noninferiority of 3RP-NF over HEP-NF, assuming genuinely no (zero) difference between treatments in the maintenance of any change from before baseline to after treatment.

We used SAS, version 9.4 (SAS Institute Inc), for analyses. We used intention-to-treat principles. We applied a shared-baseline, linear mixed model with completely unstructured covariance (selected through examination of model fit indices) among up to 4 repeated measurements (baseline, after treatment, and 6- and 12-month follow-up) and unstructured random visit-specific effects by session cohort to examine treatment effects on the primary and secondary outcomes. We ran a series of contrasts to assess the specific hypotheses about the within-group and between-group differences in the outcome variables at different time points. The model included all available data, including data from participants with incomplete evaluations and those lost to follow-up. The estimated covariance among repeated measurements implicitly imputes missing data and yields unbiased estimates under a missing-at-random assumption. The shared-baseline assumption represents the actual state of the population before randomization. It has the advantage of adjusting for chance differences at baseline in the same way as analysis of covariance.^22^

Treatment-dependent differences on changes from baseline to after treatment and to 6- and 12-month follow-up, and persistence of a benefit after treatment to the 6- and 12-month follow-up for each outcome were estimated from linear contrasts of least-square means and reported as point estimates and their unadjusted 95% CIs. Superiority of 3RP-NF at baseline to after treatment was based on a 2-tailed *P* < .025 to account for 2 primary outcomes. A noninferiority criterion for the persistence of benefit was prespecified (lower 1-sided 95% confidence bound for a given primary outcome less than the MCID of 6.25 units in favor of HEP-NF). Still, we present those results because the data demonstrated that 3RP-NF was superior to HEP-NF in maintaining benefits. We calculated the model-based effect size (ES) estimate (standardized mean difference for each comparison).^23,24,25^ We divided the model-derived mean difference estimates by the pooled within-individual variations of each outcome at baseline. This ES index is equivalent to the Cohen *d* in RCTs.^23,24,25^

We performed sensitivity analyses. We tested treatment-dependent differences in the change of score from baseline to after treatment and at 6- and 12-month follow-up using the Wilcoxon rank-sum test to relax the parametric assumptions of the mixed model. We investigated more parsimonious covariance structures using random participant-specific intercepts, slopes, and quadratic terms (ie, growth curve analysis).

Primary inference of efficacy was based on the estimated mean difference between 3RP-NF and HEP-NF in change from baseline to after treatment for primary outcomes of physical health and psychological QOL scores, testing each at a 2-tailed *P* < .025 to maintain an overall type I error rate of 5%. Given the lack of significance for these tests, unadjusted *P* values and 95% CIs are reported for comparisons with other time points and end points without claims of statistical significance.

## Results

### Study Flow

**Figure 1.**
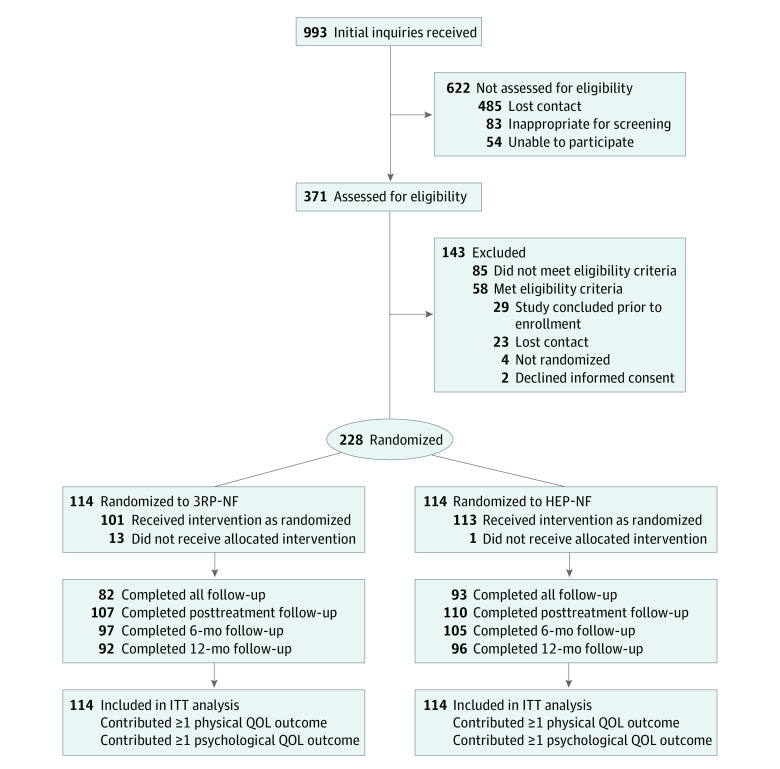
Study Flow 3RP-NF indicates Relaxation Response Resiliency Program for Neurofibromatosis; HEP-NF, Health Enhancement Program for Neurofibromatosis; ITT, intention-to-treat; and QOL, quality of life.

Of 371 individuals, 228 (mean [SD] age, 42.7 [14.5] years; 170 women [75%]; 84% White individuals) (Table 1) were eligible and randomized to 3RP-NF (114 [50%]) or HEP-NF (114 [50%]) (Figure 1). There were no differences between completers and noncompleters at each time point or between 3RP-NF and HEP-NF groups in baseline and demographic variables.

**Table 1.** Descriptive Statistics for the Sample

Characteristic	No. (%)	
	3RP-NF (n = 114)	HEP-NF (n = 114)
NF type		
NF1	83 (73)	83 (73)
NF2	16 (14)	16 (14)
Schwannomatosis	15 (13)	15 (13)
Age, mean (SD), y	42.7 (14.3)	42.8 (14.8)
Sex		
Female	83 (73)	87 (77)
Male	31 (27)	26 (23)
Race		
American Indian or Alaska Native	0	1 (1)
Asian	3 (3)	4 (4)
Black or African American	5 (4)	3 (3)
White or Caucasian	93 (82)	98 (86)
>1 Race	10 (9)	4 (4)
Choose not to answer	3 (3)	4 (4)
Ethnicity		
Hispanic or Latino or Latina	5 (4)	9 (8)
Not Hispanic or Latino or Latina	109 (96)	99 (87)
Choose not to answer	0	6 (5)
Marital status		
Married	46 (40)	52 (46)
Living with someone in a committed relationship	7 (6)	8 (7)
Single	48 (42)	42 (37)
Separated	1 (1)	1 (1)
Divorced	10 (9)	6 (5)
Widowed	2 (2)	3 (3)
Choose not to answer	0	2 (2)
Education, mean (SD), y	14.5 (3.7)	14.3 (4.5)
Learning disability		
Yes, I received a diagnosis of one	25 (22)	28 (25)
I think so, I was never formally diagnosed	24 (21)	18 (16)
No	51 (45)	58 (51)
I do not know	13 (11)	9 (8)
Choose not to answer	1 (1)	1 (1)

Abbreviations: 3RP-NF, Relaxation Response Resiliency Program for Neurofibromatosis; HEP-NF, Health Enhancement Program for Neurofibromatosis; NF, neurofibromatosis.

### Sample Characteristics

A total of 166 participants (73%) had NF1, 32 (14%) had NF2, and 30 (13%) had schwannomatosis; 217 attended 6 or more of 8 sessions and provided posttest data (Table 1). Baseline QOL in both the 3RP-NF and HEP-NF groups was poor across all domains.^26^ Mean (SD) values on all QOL domains are presented by group in Table 2.

**Table 2.** Standardized World Health Organization Quality of Life Brief Version Values at Baseline, After Treatment, and at 6- and 12-Month Follow-up

Outcome measure	Group	At baseline		After treatment		At 6 mo		At 12 mo	
		No.	Mean (SD)	No.	Mean (SD)	No.	Mean (SD)	No.	Mean (SD)
Physical health quality of life	3RP-NF	114	57.7 (19.4)	108	62.7 (20.1)	97	63.8 (20.7)	92	64.4 (22.5)
	HEP-NF	114	57.5 (20.4)	110	64.2 (17.9)	105	62.5 (19.4)	97	61.0 (21.1)
Psychological quality of life	3RP-NF	114	50.5 (17.2)	107	58.7 (16.7)	96	58.9 (19.4)	91	59.4 (20.7)
	HEP-NF	114	51.6 (17.4)	109	60.4 (15.5)	105	58.2 (17.3)	96	56.4 (16.8)
Social relationships quality of life	3RP-NF	112	57.3 (22.6)	104	63.8 (24.5)	94	64.0 (24.6)	89	67.1 (23.7)
	HEP-NF	114	58.3 (22.5)	105	65.4 (20.0)	105	63.5 (21.8)	94	61.7 (23.5)
Environmental quality of life	3RP-NF	114	70.3 (16.4)	107	73.4 (16.0)	97	75.3 (15.7)	92	78.0 (16.1)
	HEP-NF	114	67.5 (16.5)	109	73.9 (14.0)	104	72.9 (15.7)	97	72.3 (15.3)

Abbreviations: 3RP-NF, Relaxation Response Resiliency Program for Neurofibromatosis; HEP-NF, Health Enhancement Program for Neurofibromatosis.

 A total of 82 participants (72%) in the 3RP-NF group completed all follow-ups vs 93 participants (82%) in the HEP-NF group (Figure 1). The noncompletion rates for each subscale of QOL were generally comparable across intervention conditions at different time points (3RP-NF group: 0% [0 of 114] to 2% [2 of 114] for different dimensions of QOL at baseline, 5% [6 of 114] to 9% [10 of 114] after treatment, 15% [17 of 114] to 17% [20 of 114] at 6 months, and 19% [22 of 114] to 22% [25 of 114] at 12 months; and HEP-NF group: 0% [0 of 114] at baseline, 3% [4 of 114] to 8% [9 of 114] after treatment, 8% [9 of 114] to 9% [10 of 114] at 6 months, and 15% [17 of 114] to 18% [20 of 114] at 12 months). Overall, fewer than 4% of the questions were missed on questionnaires. Missingness at the subscale level was less than 1%. The linear mixed modeling approach addresses the missing data issue at the subscale level.^27^

### Primary QOL Outcomes

**Figure 2.**
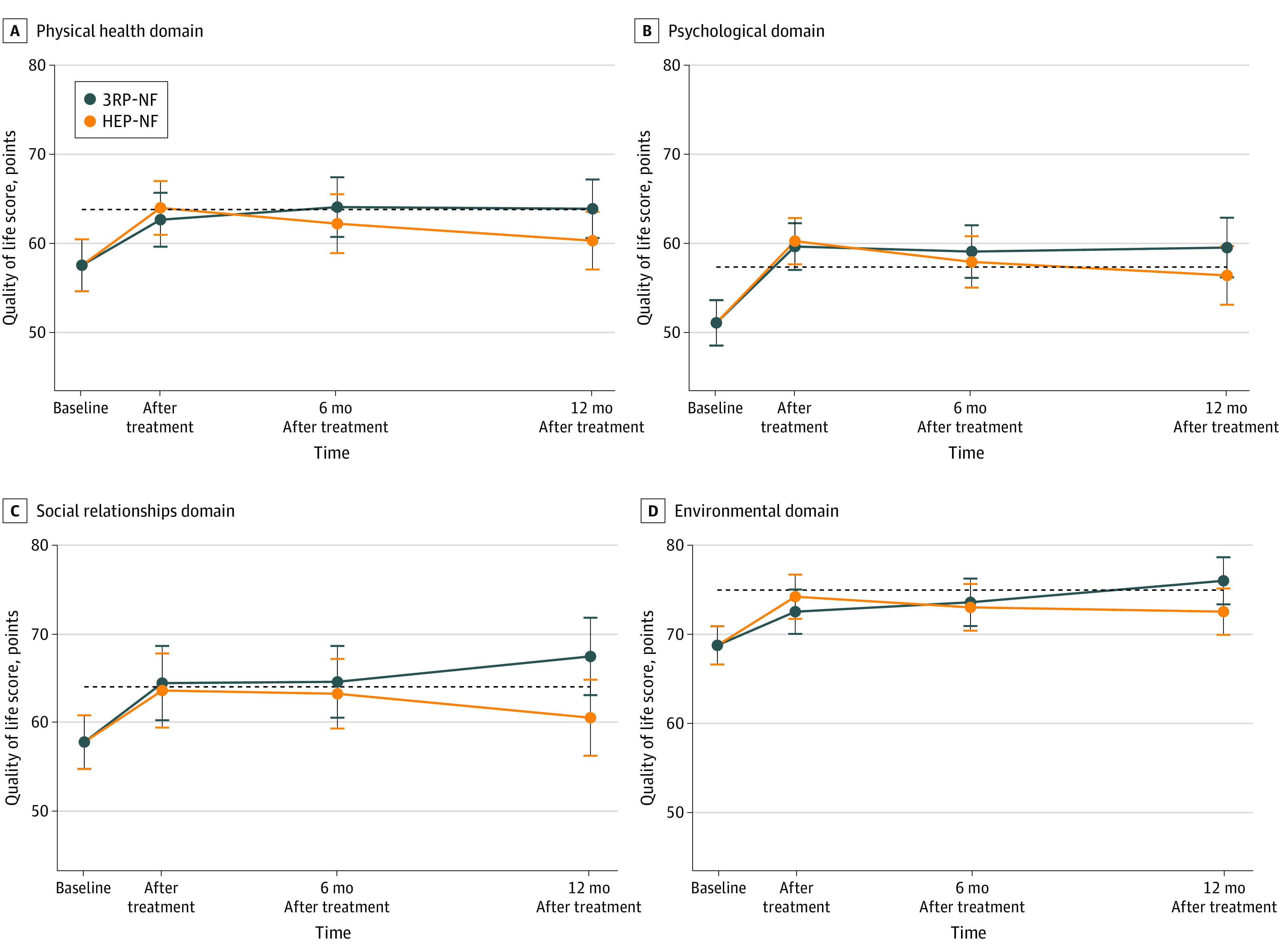
World Health Organization Quality of Life Brief Version Domain Scores Changes in mean scores at baseline, after treatment, and at 6- and 12-month follow-up. 3RP-NF indicates Relaxation Response Resiliency Program for Neurofibromatosis; HEP-NF, Health Enhancement Program for Neurofibromatosis.

Participants in both the 3RP-NF and HEP-NF groups experienced improvement in the physical health QOL score (3RP-NF, 5.1; 95% CI, 3.2-7.0; *P* < .001; HEP-NF, 6.4; 95% CI, 4.6-8.3; *P* < .001) and psychological QOL score (3RP-NF, 8.5; 95% CI, 6.4-10.7; *P* < .001; HEP-NF, 9.2; 95% CI, 7.1-11.2; *P* < .001) from baseline to after treatment (Figure 2). Improvement was clinically meaningful (above the MCID) for physical health QOL (HEP-NF) and psychological QOL (HEP-NF and 3RP-NF). The magnitude of improvement from baseline to after the test was similar between groups (physical health QOL score, −1.3; 95% CI, −3.9 to 1.2; *P* = .31; psychological QOL score, −0.6; 95% CI, −3.3 to 2.1; *P* = .67) (eTable 1 in Supplement 2). Improvements in both outcomes remained at 12 months after baseline in both groups, but the size of improvement decreased for those in the HEP-NF group and were sustained for those in the 3RP-NF group. By 12 months after baseline, improvements in both outcomes were clinically significant only for participants receiving the 3RP-NF (physical health QOL score: 3RP-NF, 6.3; HEP-NF, 2.8; difference, 3.6; psychological QOL score: 3RP-NF, 8.4; HEP-NF, 5.3; difference, 3.1). The between-group comparison of baseline to 12-month improvement in physical health QOL score favored the 3RP-NF group (3.6; 95% CI, 0.5-6.6; *P* = .02; ES = 0.2). The between-group comparisons of the durability of treatment effects from after treatment to 12-month follow-up favored the 3RP-NF group for both physical health QOL score (4.9; 95% CI, 2.1-7.7; *P* = .001; ES = 0.3; lower 1-sided 95% confidence bound = 2.5) and psychological QOL score (3.7; 95% CI, 0.2-7.6; *P* = .06; ES = 0.2; lower 1-sided 95% confidence bound = 0.4) (eTable 2 in Supplement 2).

For physical health QOL, treatment response rates for 3RP-NF vs HEP-NF were 46% (51 of 110) vs 48% (53 of 110) after treatment, 53% (54 of 102) vs 43% (46 of 107) at 6 months, and 55% (46 of 84) vs 38% (35 of 92) at 12 months. Only at 12 months were these rates significantly in favor of the 3RP-NF intervention (χ^2^ = 4.94; *P* = .03). For psychological QOL, treatment response rates for 3RP-NF vs HEP-NF were 56% (62 of 110) vs 59% (65 of 110) after treatment, 50% (50 of 101) vs 51% (54 of 107) at 6 months, and 54% (45 of 83) vs 48% (45 of 93) at 12 months. None of these rates were significantly different across groups.

### Secondary QOL Outcomes

Participants in both the 3RP-NF and HEP-NF groups experienced improvement in the social relations QOL score (3RP-NF, 6.7; 95% CI, 3.1-10.2; *P* = .001; ES = 0.3; HEP-NF, 5.8; 95% CI, 2.3-9.3; *P* = .001; ES = 0.3) and the environmental QOL score (3RP-NF, 3.7; 95% CI, 1.6-5.9; *P* = .001; ES = 0.2; HEP-NF, 5.5; 95% CI, 3.3-7.6; *P* < .001; ES = 0.3) from baseline to after treatment (eTable 1 in Supplement 2). Improvement was clinically meaningful (above the MCID) only for the social relationships QOL score for those who participated in the 3RP-NF. The improvements from baseline to after the test were similar between treatments. Improvement in both outcomes from baseline remained at 12 months in both groups, except for the social relationships QOL score in the HEP-NF group. The between-group comparison of baseline to 12-month follow-up showed improvements in both outcomes, favoring the 3RP-NF group (social relationships QOL score, 6.9; 95% CI, 1.2-12.7; *P* = .02; ES = 0.3; environmental QOL score, 3.5; 95% CI, 0.4-6.5; *P* = .02; ES = 0.2); these improvements were clinically meaningful. Although both groups maintained improvements from after treatment, the between-group tests of the durability of treatment effects from after treatment to 12 months was in favor of the 3RP-NF group, which showed an additional increase in the environmental QOL score (environmental QOL score, 5.2; 95% CI, 2.1-8.3; *P* = .001; ES = 0.3; lower 1-sided 95% confidence bound = 2.6; social relationships QOL score, 6.1; 95% CI, 1.6-10.6; *P* = .008; ES = 0.3; lower 1-sided 95% confidence bound = 2.3) (eTable 2 in Supplement 2).

For social relationships QOL, treatment response rates for 3RP-NF vs HEP-NF were 50% (53 of 106) vs 48% (51 of 106) after treatment, 56% (55 of 98) vs 43% (46 of 107) at 6 months, and 59% (47 of 80) vs 44% (40 of 91) at 12 months. Only the differences in rates at 12 months favored the 3RP-NF intervention (χ^2^ = 3.72; *P* = .05). For environmental QOL, treatment response rates for 3RP-NF vs HEP-NF were 42% (46 of 110) vs 55% (60 of 110) after treatment, 51% (52 of 102) vs 49% (52 of 106) at 6 months, and 51% (43 of 84) vs 48% (45 of 92) at 12 months. None of these rates were significantly different across interventions. In sensitivity analysis, the nonparametric Wilcoxon rank-sum test led to the same inference for the changes in the primary and secondary outcomes from baseline to after treatment and at 6- and 12-month follow-up.

## Discussion

To our knowledge, this is the first fully powered RCT of a psychosocial intervention for NF. Participation in the 3RP-NF and HEP-NF was associated with similar improvements from baseline to after treatment in primary (physical health and psychological QOL) and secondary (social relationships and environmental QOL) outcomes (small to medium ESs). Improvements persisted through 12 months, but the magnitude of improvement decreased for those in the HEP-NF group while it was sustained (primary outcomes: physical health and psychological QOL; and secondary outcome: social relationships QOL) or increased (secondary outcome: environmental QOL) for those in the 3RP-NF group. The 3RP-NF participants demonstrated within-group improvements in all primary and secondary QOL domains from baseline to 12 months, which were clinically meaningful. Participation in the 3RP-NF intervention was associated with substantially more improvement from baseline to 12 months than with the HEP-NF for all outcomes (small to medium ESs), except for the primary outcome of psychological QOL, where improvement was similar. Results demonstrate that the 3RP-NF intervention has durable benefit that exceeds that of health education. Results were similar when accounting for treatment responders based on the MCID developed for cancer.

Although we found no between-group differences from baseline to after the test in improvement in QOL domains, the magnitude of differences widened over time such that between-group differences from baseline to 12 months favored the 3RP-NF intervention. This outcome was due to either continued improvement in the 3RP-NF group (secondary outcomes) or deterioration in the HEP-NF group (primary outcomes; small to medium ESs). Only 3RP-NF participants showed improvements from baseline to 12 months that were also clinically meaningful. These findings highlight the effect of support and attention from the therapist in both groups. The continued improvement in QOL in the 3RP-NF group after the intervention ended and support from group members and therapist was removed highlights the importance of the mind-body skills taught in the 3RP-NF. It is also possible that the 3RP-NF group demonstrated improvement through 12 months because more than 8 weeks are needed to translate into improvement in QOL. This hypothesis is best illustrated for the secondary outcome of environmental QOL, which had the steepest slope of increase in the 3RP-NF group after the intervention ended. This outcome assessed access to financial resources, transportation, employment, and housing, which require time to achieve. Future studies should explore mechanisms of improvement through 3RP-NF skills to formally test this hypothesis.

### Limitations and Strengths

This study has some limitations. We included predominantly White, educated women; outcomes may not be generalizable to other groups. We also used an MCID developed for patients with cancer. This study also has some strengths. It used a geographically diverse sample and conservative randomization scheme; implemented a shared baseline assumption; and used the highest level of control for therapist attention, rigorous fidelity, and a QOL measure recommended by the REiNS International Collaboration.

## Conclusions

Findings of this RCT are consistent with our prior pilot study.^12^ The 3RP-NF intervention showed sustained and clinically meaningful improvement in all QOL domains, which increased through 12 months of follow-up. Results support the implementation and dissemination of the 3RP-NF.

## Notes

Supplement 1.
Trial Protocol

Supplement 2. **eAppendix 1.** Code, Equations, and Descriptions of the Linear Mixed Models **eTable 1.** Within- and Between-Group Differences in Quality of Life Domains From Baseline to Post-Treatment, Post 6-Months, and Post 12-Months **eTable 2.** Durability of Treatment Effects Measured by Within- and Between-Group Differences in Quality of Life Domains From Post-Treatment to Follow-up **eAppendix 2.** Projected Timeline for Patient Groups and Data Collection (Simplified)
eReferences
**eAppendix 3.** Details on The 3RP-NF Intervention

Supplement 3.
Data Sharing Statement
