# Supplementary material for: Effect of Mind-Body Skills Training on Quality of Life for Geographically Diverse Adults With Neurofibromatosis: A Fully Remote Randomized Clinical Trial
Source: JAMA Netw Open. 2023 Jun 28;6(6):e2320599. doi: 10.1001/jamanetworkopen.2023.20599 (PMC10308247; doi:10.1001/jamanetworkopen.2023.20599)
Supplement: Supplement 1. — Trial Protocol [file jamanetwopen-e2320599-s001.pdf]

A) eProtocol: Trial Protocol.

PARTNERS HUMAN RESEARCH COMMITTEE DETAILED PROTOCOL

Version Date:6.12.20

I. BACKGROUND AND SIGNIFICANCE

Neurofibromatoses are prevalent and incurable conditions. The neurofibromatoses, including neurofibromatosis 1 (NF1), neurofibromatosis 2 (NF2), and schwannomatosis, are a group of genetically distinct disorders of the nervous system unified by the predisposition to nerve sheath tumors. These histologically benign tumors can occur anywhere in the body and often cause significant morbidity including disfiguring cutaneous tumors (NF1); complete hearing loss, facial weakness, and poor gait (NF2); and chronic disabling pain (schwannomatosis). In addition to the increased risk of benign tumors, patients with these genetic syndromes are at higher risk for malignant tumors and can develop non-tumor manifestations that affect the nervous system, eyes, and skin. Currently there is no cure for NF, with symptom management provided by surgery and palliative measures as the primary means of treatment. Neurofibromatosis is the most common genetic neurological disorder that is caused by a single gene, and affects more than 100,000 people in the United States. The recognized prevalence is on the rise due to increased awareness and diagnostic techniques.

Neurofibromatoses are associated with decreased quality of life, increased stress and psychological distress, and increased medical care utilization. A recent systematic review led by the PI (Vranceanu) has shown that patients with NF have lower quality of life compared to the general population.[1] Patients with NF also have significantly more symptoms of depression and anxiety, higher levels of perceived stress, lower levels of self-esteem, and more pain as compared with general population norms.[2,3] The level of emotional distress in patients with NF is comparable to that of patients with cancer and coronary heart disease.[3] Furthermore, research has shown that there is no association between severity of NF and emotional functioning[3,4] suggesting that psychosocial factors are at least as important as severity of disease in determining patients' experience of NF. Despite their distinct pathophysiology, the psychosocial profile and emotional functioning are similar in patients with NF1, NF2 and schwannomatosis.[1,3] Among all patients with NF, those with more difficulty coping and depression by self-report also undergo more medical visits,[3,4] a finding that is common in other acute and chronic conditions.[5,6]

Current treatment of neurofibromatoses is biomedical. The current health care model for neurofibromatoses is almost entirely biomedical. In this model, health care providers have focused on identifying various manifestations of NF (e.g., nerve sheath tumors, learning disability, pseudoarthrosis) and providing medical treatments. This model of care is incomplete, as it does not address the aforementioned psychosocial factors associated with NF, which are impacting quality of life and efficacy of medical treatments. Until now, all clinical trials in patients with NF have been focused on drug testing and other medical treatments.

Psychosocial treatments are efficacious as adjuncts to medical treatments. Within the past decade, the care of patients with medical illnesses (e.g., diabetes, chronic pain, cancer) has transitioned from biomedical to biopsychosocial, where evidence-based mind-body treatments are integrated within medical care.[7] Research on such biopsychosocial models has shown that they not only improve quality of life and enhance outcomes of medical treatments, including surgeries, but also reduce medical care utilization and cost.[8] In spite of all the aforementioned factors, biopsychosocial models of care have not yet been studied in patients with NF. This represents an unexplored and potentially cost-effective opportunity to improve quality of life, buttress the efficacy of medical treatments, and decrease cost of care for patients with NF.

Given the lack of cure and the fact that medical treatments are limited in their ability to eliminate the physical effects and medical complications in patients with NF, psychosocial factors represent one sure way to increase quality of life in this population. While it is important for healthcare providers and researchers to continue striving to find effective biological treatments, and while quality of life is an important outcome measure to include in drug/medical clinical trials, we must also attend to factors that are potentially and more readily modifiable, such as psychosocial factors. Interventions aimed at enhancing quality of life by adjusting psychosocial factors are efficacious, and already implemented as part of usual care for many chronic conditions. We have shown that there is interest in a mind-body intervention in patients with NF and that such an intervention significantly improves quality of life and other psychosocial variables.[9,10]

Psychosocial treatments delivered via videoconferencing are as effective as face-to-face treatments for many chronic illnesses. Psychological interventions have traditionally been delivered in person, but researchers have recently begun to explore the efficacy of delivering psychosocial interventions online or using videoconferencing software such as Skype. Skype has also been integrated in clinical practices in several parts of the country; for example, University of California at San Francisco (UCSF) Children's Hospital and Medical Centers have partnered with Skype to facilitate communication between patients and caretakers. Further, the National Institutes of Health is currently funding several clinical trials that use Skype with various populations from dementia to HIV. Skype has also been used in a variety of published clinical studies.[11,12,13] More recently, HIPPA approved and 100% secure videoconferencing platforms have been developed and implemented in clinical care in major medical settings. One of such platform, Vidyo, is currently used to provide psychological care for patients at the Massachusetts General Hospital. The platform has received high scores on patient satisfaction from patients of various ethnicities, educational levels, cognitive functioning and psychiatric conditions (data available from MGH Telepsychiatry). Further, psychological care using Vidyo is reimbursable now by major insurance companies.

Videoconferencing is a promising avenue for research with NF patients given the need to deliver care to a geographically dispersed population, with patients who are unable to travel to specialized clinics due to financial, time, and travel considerations. In particular, videoconference interventions present an attractive option to balance practical participation issues with a desire to maintain close contact and a group atmosphere within an intervention. Interventions delivered via videoconferencing have been found to be as efficacious or more efficacious than face-to-face interventions in many conditions, including Crohn's disease,[11] cancer,[14] and smoking cessation.[15] Many patients with NF experience these practical burdens when seeking care, given that NF is a rare disease, there are few specialty NF clinics within the United States, and many patients must travel long distances to reach their medical appointments. At the Neurofibromatosis Clinic at Massachusetts General Hospital (MGH), for example, 56% of patients live more than 30 miles from clinic, and 12% live more than 250 miles from clinic. In addition, many patients have difficulty coming to clinic frequently due to work restrictions and limited mobility.

The Relaxation Response Resiliency Program (3RP) is a novel mind-body intervention.

The Relaxation Response Resiliency Program (3RP)[16] is a comprehensive, multimodal treatment that was designed to increase quality of life, resiliency and ability to cope with medical symptoms and stress. This program is rooted in the elicitation of the relaxation response (RR), which is a state of calm and increased awareness. Four decades of empirical studies by Herbert Benson and others have characterized the effects of eliciting the relaxation response on genomic, structural, physiologic, psychological, and functional outcomes.[17, 18, 19, 20, 21, 22, 23]

The 3RP combines elicitation of the RR with evidence-based findings on stress/symptom management, cognitive behavioral theory and with the evolving field of positive emotions. The 3RP has 3 three core components: 1) Relaxation Response-elicitation strategies; 2) Stress/medical symptoms Appraisal and Coping; and 3) Growth Enhancement. Blending of these three core elements creates a unique adaptive strategy to coping with stress and medical symptoms, thus increasing one's resiliency and quality of life. In this model, patients learn to 1) adapt to stress/medical symptoms by eliciting the RR; 2) generate adaptive thoughts to counter negative, stress-activating thoughts; 3) engage in lifestyle behaviors that buffer the stress response and promote the RR; 4) experience pleasure and appreciation in daily, meaningful pursuits; and 5) engage in pro-social, empathic, and pleasant behaviors. Mastery and practice of these skills is thought to help patients increase ability to cope with medical symptoms, improve mood (depression and anxiety), improve mindfulness and gratitude, leading to increased quality of life. The 3RP model is depicted below:

Figure 1. The 3RP model components

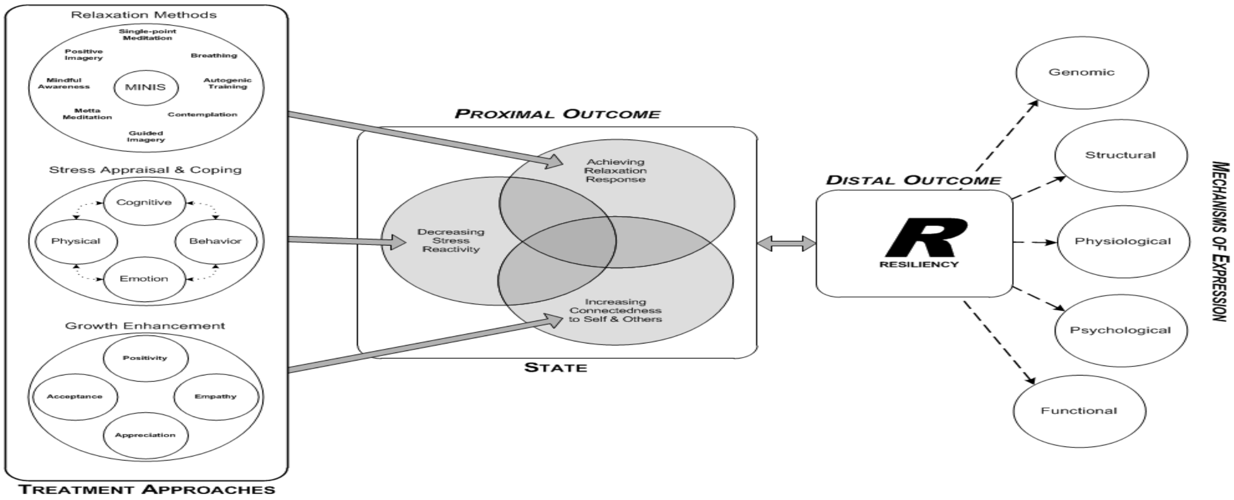

The 3RP uses a multimodal approach to introduce and reinforce new skills, including didactics, in-session activities, discussions, and weekly practice assignments (homework). The 3RP sessions reflect a purposeful integration of the three treatment approaches (relaxation methods, stress appraisal & coping, and growth enhancement). The format is an 8-week program with weekly meetings and a focus on relaxation response strategies, cognitive behavioral training, positive psychology and mind-body interactions. Each session begins with the practice of a new exercise to elicit the relaxation response. By quieting the mind and reducing distracting thoughts (e.g., achieving the relaxation response), the participant may increase absorption and retention of other skills that are introduced in the remainder of the session. Therefore, this format is reinforced throughout the 3RP. The relaxation method is then coordinated with the remaining session content. For instance, the clinician may introduce imagery of a peaceful place as a relaxation method, and then transition to didactics and exercises that focus on cognitive and behavioral skills for building a positive perspective.

Each session includes repetition of core components. First, there is a brief assessment period of the recent level of stress/medical symptoms experienced as well as one's ability to cope with these. This is followed by a check-in of the previous week's progress, RR home practice, and skill utilization. For experiential exercises, each session opens with an introduction to a new relaxation response strategy and each session closes with a "mini" relaxation technique. Please see attachment (Intervention.pdf) for session-by-session details on the 3RP adapted for patients with NF (3RP-NF).

The 3RP-NF was designed to accommodate patients at a 6th grade reading and comprehension level. To further accommodate patients with learning disabilities or other cognitive difficulties, patients are asked to have the manual with them during the Skype sessions, and to highlight the key sections (pointed out by the clinician) for further reading. The 3RP-NF was design to incorporate multiple NF specific examples, some general and others specific to each group member. Participants practice in-session skills together but also individually, thus ensuring comprehension of skills for all participants.

Preliminary studies on the 3RP/Current Clinical Use: The 3RP has evolved over the years from its inception as Medical Symptom Reduction Program to its current standardized form. The original 3RP has been tested in patients with chronic temporomandibular disorder, chronic medical illness, anxiety and depression, and among palliative care clinicians. Below we present selected empirical data on the 3RP.

1. Dusek J, Otu HH, Wohlhueter AL, Bhasin M, Zerbini LF, Joseph MG, Benson H, Libermann TA. "Genomic counter-stress changes induced by the relaxation response." 2008 July; 3(7)*:* e2576. doi: 10.1371/journal.pone.0002576.[24]

This is genomic study showing positive differences in the gene expression profiles of practitioners of relaxation response techniques when compared to healthy controls.

2. Park ER, Traeger L, Vranceanu AM, Scult M, Lerner JA, Benson H, Denninger J, Fricchione GL. The development of a patient-centered program based on the relaxation response: the Relaxation Response Resiliency Program (3RP). Psychosomatics. 2013 Mar-Apr; 54(2): 165-74.[16]

This is a review paper describing the development and theoretical underpinning of the 3RP.

3. Vranceanu AM, Shaefer JR, Saadi AF, Slawsby E, Sarin J, Scult M, Benson H, Denninger JW. The Relaxation Response Resiliency Enhancement Program in the Management of Chronic Refractory

Temporomandibular Joint Disorder: Results from a Pilot Study. Journal of Musculoskeletal Pain. 2013; 3(21): 224-230.[25]

This is a pilot showing the feasibility, acceptability and preliminary effect of the 3RP in reducing pain and improving objective functioning in patients with chronic refractory temporomandibular joint disorder.

4. Bhasin MK, Dusek JA, Chang BH, Denninger JW, Fricchione GL, Benson H, Libermann TA. Relaxation response induces temporal transcriptome changes in energy metabolism, insulin secretion and inflammatory pathways. PLoS One. 2013 May; 8(5): e62817[26]

This epigenetic study showed rapidly induced, positive changes in gene expression associated with metabolism, insulin, and inflammation in healthy novices following the 3RP.

5. Vranceanu AM, Gonzalez A, Niles H, Fricchione G, Baim M, Young A, Denninger JW, Park ER. Exploring the effectiveness of a modified comprehensive mind-body intervention for medical and psychological symptom relief. Psychosomatics. 2014; DOI:10.1016/j.psym.2014.01.005[27].

This is an effectiveness study showing improvement in psychosocial variables after the 3RP in patients with chronic medical and psychiatric illness.

6. Denninger J, Jacquart J, Miller K, Radossi A, Haime V, Macklin E, Gilburd D, Oliver MN, Mehta D, Yeung A, Fricchione G, Benson H. The effectiveness of a community-based mind body group intervention for depression & anxiety: a pilot study. J Altern Complement Med. 2014 May; 20(5): A55[28].

This is an effectiveness study showing improvement in depression and anxiety in patients undergoing the 3RP intervention.

7. Mehta D, Chittenden E, Denninger J, Haime V, Traeger L, Jackson V, Park E. Promoting resiliency among palliative care clinicians: a pilot intervention. J Altern Complement Med. 2014 May; 20(5): A102[29].

This is an open pilot that showed feasibility, acceptability and preliminary effect of the 3RP in increasing resiliency in palliative care clinicians.

8. Denninger JW, Bhasin M, Huffman J, Niles H, Creager M, Pande R, Liberman T, Fricchione G, Benson H, Zusman R. Clinical and genomic effects of a relaxation response-based mind-body intervention in stage I hypertension. J Altern Complement Med. 2014 May; 20(5): A54[30].

This study showed positive clinical and genomic effects in patients with hypertension undergoing the 3RP.

9. Kuo B, Bhasin M, Jacquart J, Scult MA, Slipp L, Riklin E, Lepoutre V, Comosa N, Norton BA, Dassatti A, Rosenblum J, Thurler AH, Surjanhata BC, Hasheminejad NH, Kagan L, Slawsby E, Rao SR, Macklin EA, Fricchione GL, Benson H, Libermann TA, Korzenik, Denninger JW. Genomic and Clinical Effects Associated with a Relaxation Response Mind-Body Intervention in Patients with Irritable Bowel Syndrome and Inflammatory Bowel Disease. PLoS ONE 2015 April; DOI:10.1371/journal.pone.0123861[31]
   This study showed improvements in psychosocial variables and genomic expression in patients suffering from IBS/IBD.
10. Miller, KM, Chad-Friedman, E, Haime, V, Mehta, DH, Lepoutre, V, Gilburd, D, Peltier-Saxe, D, Lilley, C, Benson, H, Fricchione, GL, Denninger, JW, Yeung, A. The effectiveness of a brief mind-body intervention for treating Depression in community health center patients. *Global Adv Health Med.* 2015 March; 4(2): 30-35.[32]
    This pilot study showed improvements in depression, spiritual growth, and mental health among depressed community health center patients.

2. Preliminary studies conducted by our team in support of our application: Below we detail the preliminary studies conducted by our team in support of our NF specific application: (1) a systematic review on quality of life in adults with NF1, NF2 and schwannomatosis[1] documenting the need to address psychosocial factors in this population; (2) two focus groups (15 participants total) with adult patients with NF1, NF2 and schwannomatosis – these data were used to modify the general 3RP and make it specific for patients with NF; (3) an open pilot trial of the adapted 3RP-NF delivered in person to N=20 participants (16 completers)[9] (4) a crossectional study assessing differences in psychosocial symptoms between patients with NF1, NF2 and schwannomatosis, and comparing scores to general population[2], and (5) a pilot single blind RCT of the 3RP-NF delivered via videoconferencing with Skype funded by Children's Tumor Foundation (CTF), which represents the main preliminary data for the current grant.

Study 1: Quality of life in adult patients with NF1, NF2 and Schwannomatosis; a systematic review (Vranceanu, Merker, Park & Plotkin, 2013).[1] This systematic review summarized data on quality of life in patients with NF1, NF2 and schwannomatosis and outlined predictors of quality of life. The aim of this study was to review the literature on quality of life among adult patients with NF1, NF2 and schwannomatosis, to identify the specific aspects of quality of life that were studied and reported in this population, and to identify predictors of

quality of life. Published research reports were included if they described quality of life in this population and met methodological quality according to a list of predefined criteria. Eight studies (7 in NF1, 1 in NF2, 0 in schwannomatosis), conducted between 2001 and 2013, met inclusion criteria. Patients with NF were reported to experience decreased quality of life compared to the general population. The majority of findings regarding predictors of quality of life were weak or inconclusive primarily due to the use of a variety of quality of life measures. Mind-body interventions that address quality of life domains may provide comprehensive and efficacious long-term treatment.

Study 2: Formative qualitative work informing the adaptation of the 3RP for NF patients (3RP-NF). The goals of this project were: 1) to determine the need of a mind body intervention for patients with NF, 2) to determine the level of interest in participation in the intervention, and 3) to inform the adaptation of the 3RP to 3RP-NF. We conducted two focus groups (N = 15 participants). Patients were adults with NF1, NF2 and schwannomatosis. They were all in agreement that a mind-body intervention is needed and enthusiastic about participating. Several themes emerged that were unique to this population. Theme 1: social stigmatization. Patients felt that as a group they have been ignored by psychosocial research. They noted general social isolation due to NF related symptoms including: 1) café au lait, freckling or facial tumors in NF1; 2) facial paralyses and deafness in NF2; and 3) pain in schwannomatosis. Theme 2: unique stressors. Generally, patients shared difficulty coping with the following stressors associated with NF: 1) appearance concerns: 2) uncertainty of diagnoses and prognosis (e.g., lack of cure and progression of disease); 3) multiple medical appointments including frequent tests such as MRIs; 4) symptom burden; 5) financial pressure; 6) pregnancy and concerns about transmitting NF to children; and 7) communication with medical providers and dealing with primary care doctors with little knowledge about NF. Theme 3: psychosocial comorbidities. Patients reported that depression and anxiety were main concerns, along with difficulties managing symptoms. When pain was present, difficulties coping with pain were mentioned. Many patients reported that antidepressants and/or pain medications did not relieve their symptoms and some reported a desire to decrease their narcotic medication intake**. Conclusion:** This study suggested that patients with NF are highly enthusiastic about participation in a mind body program, and helped delineated unique stressors associated with NF to inform the adaptation of the 3RP to 3RP-NF.

Study 3: In person open-pilot Relaxation Response Resiliency Program in patients with NF1, NF2 and schwannomatosis (Vranceanu, Merker, Plotkin and Park, 2014).[10] This study evaluated the feasibility, acceptability and preliminary effect of the 3RP-NF in adults with NF1, NF2 and schwannomatosis. Patients presenting to our Neurofibromatosis Clinic who reported stress and difficulties coping to their neurologist were asked to participate in the mind-body study. Those who agreed, met inclusionary/exclusionary criteria and signed the informed consent form were enrolled and asked to complete pre- and post-intervention measures via REDCap, an electronic data system. The 3RP-NF had 8 face-to-face sessions, 90 min/session. **Results:** The intervention had relatively low feasibility, with 32 out of 67 patients approached (48%) signing the informed consent form. The main reason cited for non-participation was burden of travel to the clinic. The intervention was highly acceptable, as evidenced by an 80% completion rate (16/20), and a score of 28.3 (out of 32 maximum score) on a Client Satisfaction Scale. Resiliency, quality of life, depression, stress, anxiety, and mindfulness improved significantly by paired t-tests, with effect sizes ranging from .73 to 1.33. Somatization and daytime sleepiness improved substantially but not significantly, with effect sizes of .54 and .92, respectively and p = .06. Social support and optimism improved modestly, perhaps due to high baseline scores and a ceiling effect. Exit interviews showed that patients believed the intervention was helpful and that the intervention's feasibility could be improved if delivered via videoconferencing with Skype. **Conclusions:** This study allowed us to show that the 3RP-NF is accepted by patients and has potential for efficacy in improving quality of life, and many psychosocial factors. To increase feasibility, the intervention should be delivered via videoconferencing.

Study 4: Baseline characteristics of patients with NF participating in stress management interventions. This study set out to describe comprehensively the psychosocial presentation of adults with NF who participated in a stress reduction study, and assess potential differences in psychosocial factors by NF type. Participants were patients who self referred or were referred by their NF doctor to a stress reduction intervention delivered face to face (N = 21) or on line with Skype (N = 63). Participants were women in majority (69%), Caucasian (75%), with mean age 51. Of the entire sample 57 participants had NF1, 19 had NF2 and 8 had schwannomatosis. **Results:** Scores on the PHQ-9 (M =17.10) suggested moderate to severe depression, while scores on the GAD-7 (M =14.05) suggested moderate to severe generalized anxiety. On the WHOQoLBref patients' scaled scores on environmental scale (M = 69.57) were not significantly different from general population norms. However, scores on the psychological (M =

52.91), the physical functioning (M = 64.79) and social (M = 55.15) subscales were significantly lower than those of the general population (p < .05). Scores on resilience, a construct that increases with age, were not significantly different from college students, but were significantly lower than those of older adults (p <. 05). Scores on Gratitude (M = 32.31) and emotional support (M =27.85) scales were also significantly lower than normal population scores (p<. 05). There was a great deal of variability in all pain assessments. The mean score on pain intensity (M= 4.43, range 0-10), and mean scores on PCS (M = 16.6, range 0-57) and BPI (M =19.66, range 0-65) were significantly lower than those of patients with back pain (p<. 05) and cancer pain (p <.05), respectively. There were no significant differences in any of the study measures among patients with NF1, NF2 and schwannomatosis (p>. 05), except on pain intensity, where patients with schwannomatosis reported pain scores significantly higher than patients with NF1 and NF2. In sum, patients with NF have significant psychological distress and difficulties coping compared to general population, and these difficulties are similar among patients with NF1, NF2 and schwannomatosis. This reinforces the need of psychosocial skills based interventions for this population and suggests that patients with NF1, NF2 and schwannomatosis can be grouped together as participants in such interventions.

Study 5: Vranceanu AM, Riklin E, Merker V, Macklin E, Park E, Plotkin SR. Mind body therapy via videoconferencing in patients with neurofibromatosis; An RCT. Neurology, 2016.
Overview: This was a single blind RCT pilot study of the 3RP-NF delivered via videoconferencing with Skype, in adults with NF1, NF2 and schwanommatosis, providing primary preliminary data for this application. The transition from face to face to Skype delivery of the intervention was done to improve feasibility and generalizability of the intervention. Patients were recruited through the CTF outreach via a flyer asking for interest in participation in a stress management study. Patients who met inclusionary and exclusionary criteria, including reports of difficulties coping with stress and NF symptoms at intake, were randomized to the 3RP-NF focused on teaching the elicitation of the relaxation response, adaptive thinking and positive psychology tools, or the Health Enhancement Program for NF attention placebo control (HEP-NF) focused on providing educational information on stress, NF, and lifestyle behaviors such as healthy eating, exercise, and sleep hygiene. Both groups were delivered online via videoconferencing with Skype. Participants were blind to the group assignment (intervention versus control). Participants from both groups received the same amount of support from the interventionist and other group members for the duration of the groups. All participants received a patient manual. Those in the 3RP-NF also received a CD that included mindfulness meditation exercises practiced during the group. Data was collected using the REDCap data management system. Patients in both groups completed homework. **Results**: The first 65 potential participants were screened for participation and 63 entered the study, completed consent, provided baseline and were randomized (32 intervention, 31 control). Of these, 63 completed the intervention (at least 6 sessions attended) and provided post-test data (32 in the 3RP-NF and 31 in the control). Of these, 53 provided 6 months follow up data. The abundance of inquiries, 100% post-test completion rate, and 82.5% 6-month follow-up completion rate indicates excellent feasibility and compliance. Satisfaction with participation in the program was high and similar at both post test and 6 months follow up between the 3RP-NF (4.19 and 3.92 out of 5) and the HEP-NF (4.10 and 3.79 out of 5) groups suggesting high acceptability and usability. The similar scores on satisfaction with both 3RP-NF and HEP-NF also suggest that blinding was maintained both at post test and 6 months follow up. Participants were middle-aged (mean±SD 43±years) and predominantly female (73%), college educated (60%), employed (73%), US-based (90%), and diagnosed with NF1 (71% NF1, 19% NF2, 10% schwannomatosis). There were no significant differences in baseline outcome variables between those randomized to 3RP-NF versus HEP-NF except for Psychological QoL, where patients in the HEP-NF had more impaired scores compared to those in the 3RP-NF (P < .05). This is considered a chance finding given the lack of significant baseline difference in the other 7 outcome measures. A mixed methods repeated measures ANOVA following intent to treat principles was employed for the data analyses. For primary (Physical Health QoL and Psychological QoL) and secondary (social relations QoL, environmental QoL, depression, anxiety, pain intensity for those with pain higher than 4, and pain interference for those with pain higher than 4) variables we compared the effect of the 3RP-NF versus HEP-NF on changes from own baseline to post-test, and from post-test to 6 months follow-up times using linear contrast. For primary outcomes, improvement from 3RP-NF from baseline to post test was considered clinically meaningful if the mean improvement was higher than the MCID of 6.25. For primary outcomes, durability was established if there was a less than 6.25 decrease in scores from post-test to follow up. Information on minimal clinically important difference (MCID) for the study variables does not exist in patients with NF. In patients with breast cancer, distribution- and anchor-based methods suggested an MCID of 1 on a 4-20 scale for the physical and psychological domains of the WHOQOL-100[33]. Extrapolated to the 0-100 scale used for WHOQOL-BREF subscales, this is equivalent to 6.25 units. This value was used to indicate meaningful change for the primary variables. Patients in the 3RP-NF showed greater improvement in Physical Health QoL (7.69; 95% CI: 0.29-15.10; P=.040], Psychological QoL [5.57; 95%

CI: 0.17-11.34; P=.056], Social Relations QoL [10.95; 95% CI: 1.57-20.31; P=.021], Environmental QoL [8.02; 95% CI: 2.57-13.48; P=.005], and anxiety [-2.32; 95% CI: -3.96 to 0.69; P=.006] compared to those in HEP-NF; gains maintained at follow-up. Patients in the 3RP-NF did not improve more than those in HEP-NF on depression, with both groups showing improvement. Patients in the 3RP-NF with baseline pain ≥5 of 10 showed improvement in pain intensity from baseline to post test [1.30; 95% CI: -2.26 to -.34; P=.009] with effects maintained at follow-up; this improvement was not greater than HEP-NF. There were more treatment responders in the 3RP-NF (P<.05). Please see appendix for full results from the paper currently in press.

Table 1. Unadjusted baseline, posttest and 6 months follow-up scores for outcomes.

Assessment	Baseline	Post test	Follow up
Physical Health QoL			
3RP-NF	68.53 (17.76)	76.34 (19.00)	76.34 (14.73)
HEP-NF	60.94 (21.16)	61.06 (22.21)	60.17 (22.74)
Psychological QoL			
3RP-NF	56.90 (18.34)	67.72 (17.87)	68.05 (16.03)
HEP-NF	48.12 (18.44)	53.36 (20.16)	51.53 (19.43)
Social relations QoL			
3RP-NF	52.60 (26.13)	63.28 (22.78)	59.90 (23.13)
HEP-NF	57.80 (20.51)	57.53 (22.18)	58.79 (24.16)
Environmental QoL			
3RP-NF	72.36 (20.76)	79.68 (17.87)	77.64 (17.80)
HEP-NF	66.13 (17.77)	65.42 (18.66)	66.64 (20.11)
Depression			
3RP-NF	16.06 (5.56)	13.56 (5.13)	12.98 (3.94)
HEP-NF	18.16 (5.28)	16.35 (5.26)	17.80 (7.67)
Anxiety			
3RP-NF	13.59 (3.32)	10.42 (3.15)	11.60 (2.58)
HEP-NF	14.53 (3.12)	13.68 (3.16)	14.52 (4.28)
Pain intensity (Baseline ≥5)			
3RP-NF	6.46 (1.59)	5.16 (2.83)	4.91 (2.34)
HEP-NF	6.84 (1.42)	6.67 (1.94)	7.09 (1.62)
Pain Interference (Baseline ≥5)			
3RP-NF	3.13 (2.52)	2.88 (2.68)	1.49 (1.57)
HEP-NF	5.29 (2.17)	5.18 (2.23)	5.12 (2.42)

Note: Values are expressed as Means and SDs

Figure 2: Improvements in Physical QoL throughout the study. Average Physical Health QoL in patients in 3RP-NF and HEP-NF at baseline, post test and 6 months follow up.

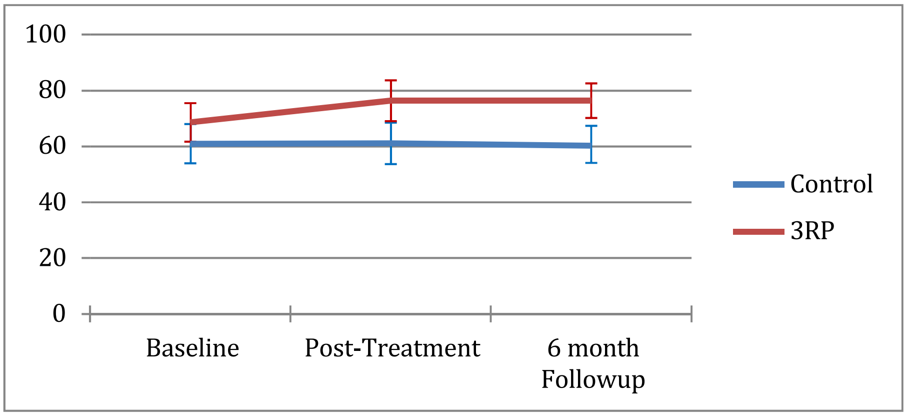

Figure 3: Improvements in Psychological QoL throughout the study. Average Psychological QoL in patients in 3RP-NF and HEP-NF at baseline, post test and 6 months follow up.

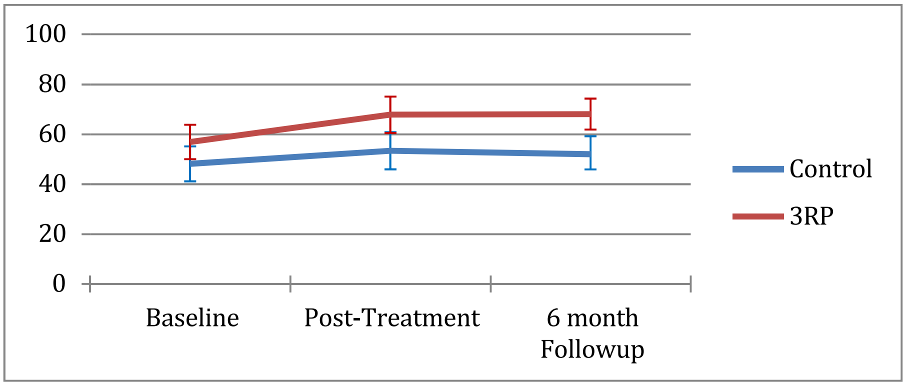

Conclusions: The 3RP-NF delivered via videoconferencing was highly feasible and accepted by patients, and resulted in sustained improvement in QoL. This study provides Class II evidence that for patients with neurofibromatosis a mind body program is superior to an attention placebo control in improving quality of life, and provides strong pilot data for a fully powered RCT to test whether effects of the intervention maintain with a more heterogenous population, and intervention delivered by different clinicians. Further, a larger sample size is required to fully understand whether the intervention if efficacious and durable for depression and pain interference. The fully powered RCT would also allow us to explore mechanisms of improvement through the use of mediators and moderators (see aims).

II. SPECIFIC AIMS

The present proposal is based on qualitative formative work, an open pilot of the face-to-face 3RP-NF, and a pilot single blinded RCT of the 3RP-NF versus HEP-NF, both delivered via videoconferencing with Skype showing feasibility, acceptability, preliminary efficacy and durability. The objective of the study is to test, within a single blinded fully powered RCT, the efficacy of the 3RP-NF as compared to HEP-NF (attention control) in improving co-primary (physical and psychological quality of life) and secondary (social quality of life, environmental quality of life, depression, anxiety, pain intensity and pain interference) outcomes. **Specific aims:**

1. **Aim 1:** to determine the efficacy of 3RP-NF in comparison to an active control HLI on the co-primary outcomes: physical health QOL and psychological health QOL among patients with NF1, NF2 and schwannomatosis. Secondary outcomes are: social relationships QoL, environment QoL, depression, anxiety, pain, pain interference, gratitude, optimism, mindfulness, coping, social support, empathy and stress. Assessments will be conducted at baseline, post-intervention (8 weeks, primary time point), 6 and 12 months follow-ups (secondary time points). Both intervention and control are delivered via live video with Zoom.

Hypotheses: 3RP-NF will be more efficacious in reducing primary and secondary outcomes at post-intervention, and effects will be maintained at 6 and 12 months follow-ups, compared to HEP-NF.

2. **Aim 2:** to examine the degree to which treatment-dependent improvements in the primary outcomes are mediated by improvements in depression, anxiety, pain intensity, pain interference, gratitude, optimism, mindfulness, coping, social support, empathy and stress (conceptual mediators) and modified by NF type, age, race/ethnicity, learning disability and education level.
   Hypotheses: Treatment-dependent improvements in the secondary outcomes depicted in the figure below will mediate benefits from 3RP-NR for the primary outcomes. The moderators NF type, age, and race/ethnicity will be associated with differential benefit from 3RP-NF for the primary outcomes.

III. SUBJECT SELECTION

Participants will be N = 224 adult patients (18 or older) with a diagnosis of NF1, NF2 or schwannomatosis. We will use a stratified randomization to ensure that equal numbers of patients with NF1, NF2 and Schwannomatosis will be assigned to the 3RP-NF and HEP-NF. Patients will be recruited from two sources: 1) the

Neurofibromatosis clinic at MGH (900 visits/year in 2014) and 2) the Children's Tumor Foundation (CTF). Our IRB approved flyers will be posted in various areas of the hospital and emailed periodically to patients in the NF registry via CTF, which includes local (within the United States) and international (outside of the United States) patients. We have used this method of recruitment successfully in our preliminary clinical trial (Vranceanu PI, funding from CTF). CTF has committed their support to recruitment, should this study be funded), and DoD has allowed for recruitment of patients within and outside of the United States. This method of recruitment is advantageous because it has allowed us to recruit patients from across the U.S. and Canada, thus fostering generalizability of results. We do not anticipate any difficulties recruiting, based on our success with working with this population. However, if difficulties occur, we plan to expand our recruitment to other U.S. NF centers with whom we have collaborations, including Children's Hospital in Boston and Johns Hopkins NF Clinic. We will also be recruiting by posting our study on Partners Clinical Trials.

Recruitment will also occur through the Research Patient Database Registry (RPDR). The RPDR is a centralized clinical data registry that gathers data from various hospital legacy systems and stores it in one place. Researchers access the data using the RPDR online Query Tool. They may query the RPDR data for aggregate totals, and with proper IRB approval, obtain medical record data. The RPDR ensures the security of patient information by controlling and auditing the distribution of patient data within the guidelines of the IRB and with the use of several built-in, automated security measures. To identify potentially eligible patients:

1) A RPDR query will be performed to identify those patients with chronic pain diagnoses. Study staff will review the medical record to confirm potential participant eligibility and to identify their linkage to an MGH primary care physician. Access to patients' medical records will be restricted to this pre-enrollment recruitment phase.
2) Study staff then will obtain permission for initial contact from each potentially eligible patient's PCP by having providers review letters and discard ones that they do not approve.
3) For physician-approved patients, study staff will send a study introduction letter from the patient's physician (with the clinician's name at the bottom) and a study opt-out letter signed by Ana-Maria Vranceanu (PI). The letter from the PCP informs the patient that he or she is allowing the study to contact patients with chronic pain in case they are interested in learning about the study. Dr. Vranceanu's letter is an opt-out letter describing the study, the procedure to opt out of further contact, and whom to call for further information.
4) Should study staff receive no reply within 10 days, staff members will call the patient on the phone to assess interest in the study and to describe the study over the phone. If the patient remains interested, staff will confirm eligibility and assess for inclusion and exclusion criteria.

For potentially eligible patients who are enrolled in the MGH Research Options Direct to You (RODY) Program, we will send them an opt-out letter and call 10 days later to inform them about the study. RODY identifies patients who are willing to be contacted directly about research studies. Patients who have agreed to be contacted directly are identifiable through the RPDR search; each patient's RODY status is available in the demographics table included in the RPDR output.

Inclusion/Exclusion Criteria

Inclusion and exclusion criteria were selected following guidelines for psychosocial treatment development[52] and for bridging efficacy with effectiveness[53] . Women and minorities will not be excluded. Screening will be done through patient self-report either in person (when patients are recruited from the MGH NF clinic) or via Zoom (when patients are recruited through CTF).

Inclusion Criteria	Exclusion Criteria
1. Has a diagnosis of NF1, NF2 or schwannomatosis and is 18 or older.	1. Has major medical comorbidity not NF related expected to worsen in the next 12 months

2. Is capable of completing and fully understanding the informed consent process and the study procedures and assessments in English.	2. Recent (within past 3 months) change in antidepressant medication
3. At least a 6th grade self-reported reading level.	3. Recent participation in cognitive behavioral therapy or relaxation therapy (within past 3 months)
4. Self reported difficulties coping with stress and NF symptoms.	4. Has significant mental health diagnosis requiring immediate treatment (e.g., untreated bipolar disorder, psychotic disorder, active substance dependence)- by self report and observation during pre-screening.
5. Score of 6 or higher on the PSS-4.	5. Unable or unwilling to complete assessments electronically via REDCap.
	6. Unable or unwilling to participate in group videoconferencing sessions.

Justification for inclusion criteria:

1. The intervention targets adult patients with NF1, NF2 and schwannomatosis. Psychoosical Interventions for adults are different than for adolescents. We are currently conducting a preliminary RCT of the 3RP-NF adapted and simplified to the needs of adolescents, via live video.
2. This inclusion criterion will ensure that patients have a thorough understanding of what the study entails and that they are able to provide valid data.
3. The 3RP-NF and HEP-NF manuals have been written to accommodate a 6th grade and higher reading level. This was done in order to accommodate patients with learning disabilities who might have trouble understanding more complex material. Patients who do not meet this criterion (via self-report) will be excluded. During our preliminary RCT, we did not exclude any participants based on this criterion. Further, patients with learning disabilities did not express any concerns about the manual or group sessions.
4. As discussed in the Narrative section, the Relaxation Response Resiliency Program (3RP-NF) is aimed at improving physical and psychological quality of life by improving coping with stress and NF symptoms. As such, the intervention will target patients who report difficulties coping with stress and NF symptoms. The advertising materials will describe that this study is geared toward improving coping with stress and NF symptoms. Further, we will ensure that this inclusionary criterion is met during the individual Skype intake session.
5. The PSS-4 is a valid and reliable measure of stress that will be used to screen for patients who have higher than average levels of stress. By doing so, we ensure that the patients who are at the highest risk are identified and approached.

Justification for exclusion criteria:

1. The presence of a major medical comorbidity not NF related represents an additional huge stressor, which can impact reports and confound results. In our preliminary RCT we had no patients excluded due to this criterion.
2. This exclusion criterion is used to ensure that any intervention effects are due to active ingredients of the intervention, not due to an antidepressant regimen change. In our preliminary RCT we had no patients excluded due to this criterion.
3. The 3RP-NF teaches elements of Cognitive Behavioral Therapy and Relaxation training. This exclusion criterion is meant to ensure that potential gains are due to the 3RP-NF not other interventions. In our preliminary RCT we had no patients excluded due to this criterion.
4. The presence of untreated major mental illness is also an exclusion criterion. Those presenting for

participation in the study that need immediate mental health services (e.g., mania, psychotic disorders) will be referred for the appropriate level of care, if that is not already in place. This level of care will be prioritized above their participation in the study. We do not anticipate that many patients will be excluded due to this criterion. In our preliminary RCT, out of 65 participants screened, only 1 was excluded due to this criterion.

5. Because participants will reside across the U.S., electronic data capture is necessary, and we will exclude those unwilling or unable to complete measures electronically. In our preliminary RCT we had no patients excluded due to this criterion.
6. Because the intervention is delivered online via videoconferencing, we will exclude patients not willing or not able to use this medium of intervention delivery. This means that deafened patients will be excluded. The desire for generalizability to all NF patients was balanced with the need to prove the efficacy of the 3RP-NF first. Once efficacy is proven, we will implement caption tools to allow deafened patients to be part of 3RP-NF groups, as part of the dissemination efforts. In fact, we are currently conducting a preliminary RCT of an adapted version of the 3RP-NF in patients with NF2 who are deaf using live video and CART.

Recruitment

We propose to enroll 224 adults (see power analyses) with NF1, NF2 and schwannomatosis ages 18 and older during the first 3 years of the proposed 4-year project. Active recruitment will be carried out by a trained and experienced research assistant with supervision from the PI. In our current videoconferencing preliminary RCT we recruited through the Children's Tumor Foundation (CTF) NF outreach program, and have received 255 inquiries from interested participants from only 1 email. The CTF is the largest NF nonprofit organization within US. We have commitment from CTF for help with recruitment for the current study (see letter of collaboration). We will also recruit from the Neurofibromatosis clinic at MGH. Flyers will be posted in various areas of the hospital as a recruitment method. Our recruitment video will be posted on the websites of NF groups (e.g., CTF) and on NF-specific social media pages. If needed, we can access other sources of referrals including NCI via Pamela Wolters, PhD, Children's Hospital via Nicole Ulrich, MD, and John Hopkins Hospital NF Clinic via Jaishri Blakeley, MD. We could also buttress recruitment via the NF Facebook group and other available social media mediums. We will also be recruiting by posting our study on Partners Clinical Trials. The Zoom medium allows for recruitment of participants from across the US, making the results generalizable to the NF population. Enrollment and retaining of participants will be buttressed by scheduling the groups in the evenings and weekends, a strategy that allowed for 100% retention in our current preliminary RCT. In our preliminary RCT we enrolled primarily educated participants. This demographic is typically most interested in participation in research. We will make efforts to recruit demographics underrepresented in our current trial though attendance of NF forums and collaboration with CTF (see Human Subjects).

IV. SUBJECT ENROLLMENT

Participants who express interest will be emailed the study consent form and scheduled for a baseline meeting via Zoom, which includes pre-screening to ensure that participants meet the study criteria, and the consent process (for participants who meet criteria). If needed, members of the research team will contact participants by phone to assist with study-related tasks (e.g., provide technical support to facilitate use of technology). All study staff will participate in National Institute of Health required training for conduct studies that involve human subjects. MGH requires all investigators to pass the Collaborative Institutional Training Initiative (CITI) course and this will be ensured for all study staff. Training for all stuff includes but is not limited to Human Subjects, Informed Consent, Good Clinical Practice, Quality Management, Confidentiality and Reporting of Adverse Events.
<u>Justification for using Zoom in this RCT:</u> The decision to use Zoom is based on weighing security of available video conferencing technologies, recommendations by MGH, and consideration of ease of use for participants. Using the Partners Zoom license ensures an end-to-end encrypted video connection, along with the ease of setting meetings to be password-protected. Zoom offers the highest quality video connection, and the easiest installment and use requirements. We believe that Zoom is very user-friendly and will enhance the quality of this RCT through its ease of use.

If as a result of participant self-report, study staff discovery, or routine assessments, the study staff might become aware of self harm; all study staff will be trained to address these issues and the PI will be informed of all such incidences and will personally contact participants to further assess self harm and ensure participant safety. As an additional precaution and consistent with feedback from the 2015 DOD review panel, we will ask all participants

to provide contact information (phone number and email) for at least 1 friend or family member that we can contact in case of emergency. Redcap has the ability to send voice or text messages securely (through "send secure" feature on an encrypted device, and we will program it to send a text to the PI in case a patient endorses the suicidality item (#9 on the PhQ-9) at any of the 4 time points. Should this happen, the PI will immediately contact the patient and/or the friend or family member previously depicted as a safety contact by the participant, and start a safety procedure (assessment, referral, etc) to ensure the safety of the participant. So far during our Skype RCT we have not had any such situations.

V. STUDY PROCEDURES

Participants will be randomized 1:1 to 3RP-NF or HLI using a permuted-block randomization, stratified by diagnosis. Stratification ensures that an equal number of participants with NF1, NF2 and schwannomatosis are allocated to both treatments. Randomization will be generated by a computer.
We will use the same clinician/study therapist across the study conditions. Consideration was given to using different therapists for each arm to minimize potential contamination. However, observed study effects might be then attributable to clinician/therapist factors, and the study manuals are distinct. To deal with potential contamination, we will employ the audio-recording supervision and review as detailed below, including the use of checklists to document therapist fidelity to the intervention and also to prevent drift.

1.1. Experimental treatment: The Relaxation Response Resiliency Program for patients with NF (3RP-NF). The original 3RP program manual was modified to address specific needs of patients with NF, which were identified previously during 2 focus groups (N = 15 participants) and literature review. During these focus groups patients were asked open ended questions about the need for a mind body group, interest in potential participation, difficulties managing NF, and about specific stressors associated with NF1, NF2 and schwannomatosis, Specific modifications included: 1) specific skills aimed at managing NF specific stressors such as burden of medical appointments, dating and difficulties hearing; 2) restructuring NF specific thoughts such as those associated with self image, career, etc; and 3) learning acceptance techniques to address issues associated with making sense of having NF and dealing with the uncertainty of having this condition. The original 3RP manual was modified to address these changes and was used uniformly with each group. Each of the 8 sessions involves a review of previously learned skills, introducing and practicing a new relaxation response technique, and learning a specific cognitive behavioral or positive psychology based skills and applying it to specific patient specific issues. Participants receive 2 relaxation CDs to ease practice at home, as well as a patient manual. Participants are instructed in the importance of practicing the skills at home, and homework forms are collected at the beginning of each session. Further, every session includes discussions on homework completion and problem solving barriers to practice. Groups are small (5-7 participants/group) allowing the facilitator to ensure that patients understand and practice the skills taught. The 3 diagnostic categories are grouped together for the purpose of this study for several reasons. First, there are many similarities among patients with NF1, NF2 and schwannomatosis such as common stressors associated with symptoms (e.g., appearance concerns, pain, uncertainty about progression/growth of tumors, social isolations, concerns about transmission through pregnancy). Second, patients within the 3 categories, as a group, share similar psychosocial difficulties and decreased quality of life. Third, the 3RP is not aimed to target underlying diseases processes, as would a drug; rather, it is aimed at improving coping with stress and symptoms (e.g., increase resiliency) and satisfaction with life. We deal with the heterogeneity of NF by asking patients to set general and weekly practice goals that are specific to them, and teaching them to apply them to their individual goals and concerns. For example, a patient with NF1 who has facial tumors might set a goal of decreasing self-consciousness while a patient with schwannomatosis might set a goal of improving coping with pain. Both patients can benefit from relaxation response strategies as well as from cognitive behavioral and positive psychology skills to help them develop acceptance and an adaptive response to a chronic incurable condition. Lastly, this decision is based on preliminary research. We found no differences in baseline characteristics of patients with NF1, NF2 and schwannomatosis on any of the study variables, with the exception of pain intensity, which was the highest in patients with schwannomatosis[2] . Further, research on the 3RP has shown that patients with various medical and psychiatric concerns respond similarly to the mind body intervention (Gonzalez, Vranceanu, & Park, submitted).

1.2. Time-Matched Attention Placebo Active Comparison Condition: Health Enhancement Program (HEP-NF). The active comparison condition controls for the effect of "time spent", "group member support/feedback" and "interventionist support/feedback" and includes delivery of educational information on NF types and symptoms drawn from the CTF website, and standard healthy living information drawn from the Center for Disease Control recommendations and standards for health promotion (e.g., "Sleep", "Nutrition", "Healthy Weight", and "Medical appointments"). These sessions were manualized and used in our preliminary RCT that

represents the basis of this research. Such control interventions are routinely used in stringent RCTs of psychosocial interventions. The HEP-NF program consists on 8 group sessions (each session is 90 minutes) that occur concurrently with the active intervention condition 3RP-NF. The active comparison HEP-NF is conducted in the same format as the intervention condition but does not include any relaxation response, cognitive behavioral or positive psychology skills training that are reflected in the 3RP-NF. Patients in the HEP-NF receive the same attention from the study therapist as those in the 3RP-NF.

6. Protocol integrity. The integrity of the 3RP-NF and HEP-NF protocols will be assured empirically. All sessions will be audiotaped. We have developed a rating checklist for therapist adherence that includes whether the specific components of each session was in fact delivered. Adherence ratings will be completed by Dr. Park. Competence will be ensured by weekly clinical supervision using audiotapes. At least 20% of the sessions, and at least one session per week, will be reviewed prior to clinical supervision meetings, and the rating checklist will be used to give specific feedback to study therapists.

7. Strategies to avoid participant unblinding: Participants will not know whether they are assigned to the 3RP-NF intervention or the HEP-NF control. Rather, they will be informed that they will be randomized to one of 2 stress management groups, in an effort to identify which one works best in patients with NF. In order to prevent unblinding, participants will be asked to not share information discussed in the group on social media sites (e.g., Facebook groups or internet chat groups) or with other NF acquaintances for the duration of the study. We will also explain to study participants how sharing of information would potentially negatively impact the validity of study results. It is important to mention that we have evidence that the HEP-NF control is credible and patients remained generally blind, as we found no differences in scores of satisfaction with overall program at posttest or 6 month follow up. Nevertheless, we anticipate some level of disclosure of techniques from the 3RP-NF groups to the HEP-NF control groups via social media sites, which would make our findings conservative by potentially *underestimating* the true efficacy.

All study assessments will be administered online, using the REDCap system. Subjects consented in person may choose to fill out these questionnaires on-site, or to fill them out at home on a personal computer or other Internet-equipped device. Subjects consented via phone will be asked to complete the questionnaires online, at home. At study intake, participants will be given baseline psychological and behavioral assessments (see attached study questionnaire). This assessment includes demographic information and a battery of psychological questionnaires, itemized below.

Administered Pre-Intervention Only:
Demographics – covariates and moderators

Administered Pre-Intervention, Post-Intervention, 6 and 12 Month Follow-Ups:
Quality of Life (WHOQOL-BREF) physical health scale[29] – co- primary outcome.
Quality of Life (WHOQOL-BREF) psychological scale[29] – co- primary outcome.
Quality of Life (WHOQOL-BREF) social relationships scale[29] – secondary outcome.
Quality of Life (WHOQOL-BREF) environmental scale[29] – secondary outcome.
The Patient-Health Questionnaire Depression (PHQ-9)[33] – secondary outcome/mediator
The Generalized Anxiety Disorder Questionnaire (GAD-7)[34] – secondary outcome/mediator
MOS Social Support Survey – secondary outcome/mediator
The Gratitude Questionnaire Six Item Form (GQ-6) – secondary outcome/mediator
Revised Life Orientation Test (LOT-R) – secondary outcome/mediator
Measure of Current Status Part A (MOCS-A) – secondary outcome/mediator
Cognitive and Affective Mindfulness (CAM) – secondary outcome/mediator
Perceived Stress Scale 10 (PSS-10) – secondary outcome/mediator
Interpersonal Reactivity Index (IRI) Empathic Subscale – secondary outcome/mediator
PROMIS Pain Interference v8 – secondary outcome/mediator
Graded Chronic Pain Scale (GCPS) – secondary outcome/mediator

Patient Homework –weekly during intervention:
Progress Note – adherence to treatment/skill consolidations/moderator.

Administered Post-Intervention, 6 and 12 Month Follow-Ups Only:
Patient Perception of Improvement – anchor-based measure of improvement

Figure 1. Study Design.

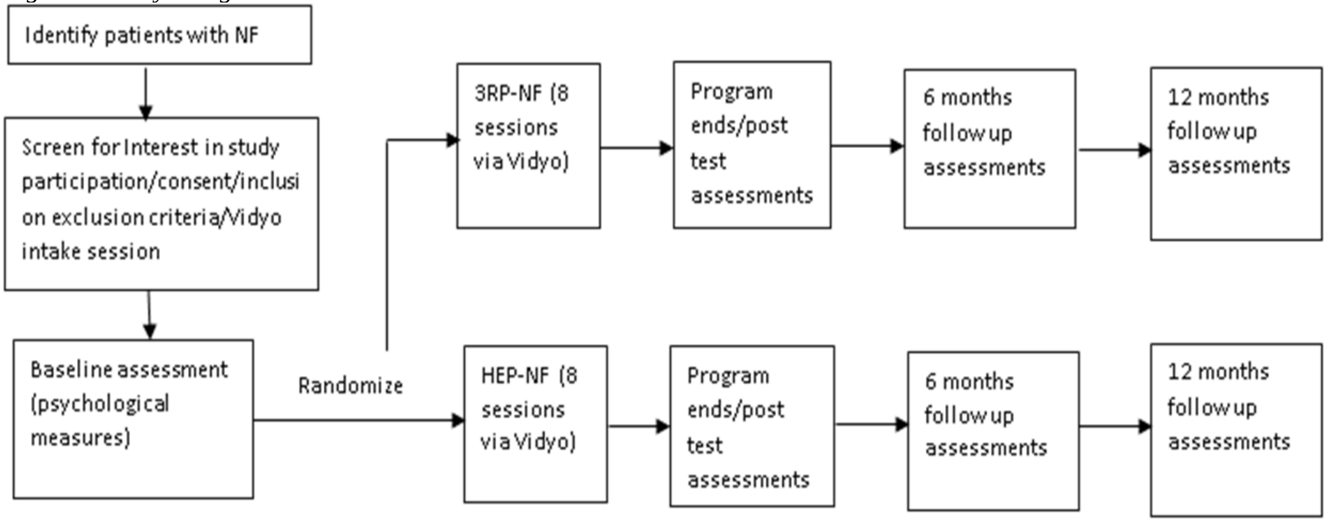

VI. BIOSTATISTIC ANALYSIS

1. Missing data: We will assess for patterns of missing data, which are expected to be low as the study uses computer-assisted questionnaire completion (REDCap), which reduces participant non-response (see how missing data is handled below). The primary anticipated reason for missing data is attrition due to loss of follow up. Based on our preliminary studies, in particular our videoconferencing RCT, we do not anticipate loss of data at time 2 (100% observed retention). However, we do anticipate some loss of data for the 6 (82.5% observed retention) and 12 months follow up. Conservatively, we estimate up to 5% loss to follow-up by post-treatment and up to 20% loss to follow-up by 12 months. Hence, we plan to enroll 224 participants to achieve at least 212 at post-treatment and 180 at 6 and 12 months. We will use mixed-model maximum-likelihood techniques for analysis to minimize bias when lost to follow-up is missing at random conditional on the observed data. Attrition effects will be evaluated by testing whether systematic differences exist between those participants who completed the research versus those who drop out. In this way we can determine the nature of the potential bias introduced by study attrition. If attrition is higher in the 3RP-NF group, multiple imputation and sensitivity analyses assuming a range of informative loss to follow-up will be investigated. Note that in our preliminary study, drop-out was more common in the placebo arm, which if it reflects lack of efficacy would yield conservative estimates of benefit from 3RP-NF.

2. Evaluating possible confounders: In the preliminary analyses we will examine the equivalence of the random assignment of groups with regard to key baseline characteristics. This will involve assessment of the treatment groups on sociodemographic characteristics, psychosocial variables and homework completion. In the unlikely event that important differences at baseline occurred by change even with randomization, we will run analyses both with and without these variables as covariates to determine whether baseline differences may account for differences in outcomes.

3. Analyses for primary aim –Specific Aim 1: Data analyses will follow a sequence that is designed to answer the primary question: Does the 3RP-NF intervention improves physical and psychosocial quality of life (co-primary outcomes), social quality of life, environmental quality of life, pain intensity, pain interference, depression and anxiety (secondary outcomes) relative to the comparison condition HEP-NF? We have already established feasibility and acceptability of the 3RP-NF, so the emphasis of this study is on establishing the efficacy (primary time point at

post-intervention) and durability (to 6 and 12 months) of the 3RP-NF. Accordingly, the primary analysis will compare changes in primary and secondary outcomes (continuous measures) from baseline to the post-, 6 months and 12 months visits between the randomized conditions. All randomized participants will be included in our primary efficacy analyses as randomized, following the intention-to-treat principle. Data will be analyzed using a shared-baseline, linear mixed model with fully unstructured covariance among repeated measures. The shared-baseline assumption reflects the true state of the population prior to randomization and has the benefit of adjusting for chance differences at baseline[48]. For each outcome, we will compare the effect of 3RP-NF vs. HEP-NF on changes from baseline to the post-test, 6 and 12 months follow-up times using linear contrasts. Our primary aim will focus on the post-treatment follow-up time. With two co-primary outcomes, we will use two-sided tests at $p < 0.025$ to declare superiority of 3RP-NF, maintaining an overall 5% type 1 error at our primary post-intervention time point. Persistence of a benefit from 3RP-NF at 6 and 12 month follow-up times will be analyzed as a non-inferiority test of durability. Non-inferiority of 3RP-NF in maintaining benefits relative to HEP-NF will be declared if the lower one-sided 95% confidence bound for a given co-primary outcome is less than 6.25 units (the estimated MCID) in favor of HEP-NF. Several sensitivity analyses will be explored using alternative models. Change scores at post-intervention, 6 months, and 12 months will be separately analyzed by Wilcoxon rank sum test to avoid any parametric assumptions about the data. More parsimonious covariance structures will be considered using random participant-specific intercepts, slopes, and quadratic terms (i.e., growth curve analysis). Baseline parameters such as NRS pain will be included to account for chance differences due to randomization and to explain sources of variation in response that are independent of treatment group.

While no information on clinically meaningful differences exists for the primary outcome measures for patients with NF, we will also use both “distribution-based” and “anchor-based” methods as criteria to determine important change.[49, 50] Consistent with “anchor based” methods, we will ask patients at post-treatment, 6 and 12 months follow ups to rate whether they were “substantially better”, “minimally better” “about the same”, “minimally worse” or “substantially worse” as compared to how they were before the intervention. These improvement ratings would thus serve as the standard with which to evaluate the importance to the patient of whatever changes in quality of life occurred during the intervention. Success of the trial will thus be estimated based on outcome score (quality of life scores) and anchor-based measures of subjective improvement. The anchor-based MCID estimates will be computed using prior methodology[33], as the mean change in the physical health and psychological health QoL among those in the “minimally better” category.

3.1. Power Consideration and Sample Size Calculations. The effective standard deviation (SD) for the change from baseline to post-treatment in QoL Physical and QoL Psychological based on a repeated-measures ANOVA of our preliminary data was 14.7 and 10.4 units, respectively. The effective SD from post-treatment to 6-month follow-up was 11.4 and 10.0 units, respectively. Based on these estimates, assuming an MCID of 6.25 units, allowing up to 5% loss to follow-up by post-treatment assessment, and testing each of the co-primary outcomes at $p < 0.025$ two-sided, the study will have 80% power for QoL Physical and 96% power for QoL Psychological. Allowing up to 20% loss to follow-up by the 6-month assessment, the study will have 99% power to declaring non-inferiority of 3RP-NF vs. HEP-NF if the true treatment-dependent difference in maintenance of any change from baseline to post-treatment is zero.

4. Analyses for specific aim 2.

4.1 Mediation: If the 3RP-NF intervention does show improvement in some or all of the primary and secondary outcomes among the sample in greater magnitude than the HEP-NF, we will explore the extent to which this relationship works through several possible mediators (some of the secondary outcomes: depression, anxiety, pain interference and pain intensity). To evaluate whether the intervention works in improving physical health QoL (as an example) thorough improved anxiety (as an example), we will conduct a product coefficients test for the effect of the intervention on anxiety as an intervening variable.[51] For this test, we will estimate 2 regression models: the first will regress changes in anxiety on the intervention to determine the effect of the intervention on anxiety, and the second will regress changes in physical QoL on changes in anxiety while adjusting for treatment arm, to assess the effect of the intervention on physical health QoL while holding changes in anxiety constant. We will then calculate the product of coefficients, 1 1, where 1 1is the coefficient of the intervention as predictor of changes in physical health QoL in the first regression model, and 1 is the coefficient of changes in anxiety as a predictor of physical health QoL changes, controlling for intervention status, in the second regression model. Statistical significance of the mediated effect would be determined by the asymmetric distribution of product test, where lower and upper confidence limits will be calculated based on the product 1 1 and a critical value[52] multiplied by the standard error of b.[51] Evidence of mediation is supported if the confidence interval does not cover zero. The

mediation effect size will be determined by the proportion of the total effect that is attributable to the mediation (i.e., the mediated effect divided by total effect).[53] This test will also be conducted separately for the assessment of increases in the other mediators. The test will also be conducted separately for the psychological health QoL outcome. This method is consistent with Baron and Kenny (1986)[54] and updated by Kraemer et al (2002) [55]; however, it extends the analysis by allowing us to test significance of the mediated effect, and quantify the magnitude of the mediation.

4.2. Moderators: The possible effect of moderators of a beneficial effect of 3RP-NF will be investigated by adding moderator (e.g., contrasting treatment response by NF1, NF2, and schwannomatosis diagnosis), moderator x treatment and moderator x treatment x visit interaction terms to the repeated-measures ANOVA described for Aim 1. Specific linear contrasts of the moderator x treatment x visit interaction terms will be used to test for differential 3RP-NF dependent benefit in improvements from baseline to post-treatment, 6 month, or 12 month follow-up that are a function of diagnosis, age, or race/ethnicity. Because in our previous work[2] we showed that in a pooled sample of patients from the current RCT and our prior pilot (N=83) there were no significant differences in baseline scores on any outcome measures we are not powering the study to detect differences by NF type. Nevertheless, by stratifying randomization by diagnosis, we will optimize our power to detect differences by NF type given the available sample size and distribution of NF types.

VII. RISKS AND DISCOMFORTS

Patients will be informed that there are no foreseeable physical risks from this research study. They will be informed that they may feel uncomfortable completing various psychological questionnaires and that they may find it time-consuming to participate in weekly 90 minute groups.

As Zoom is HIPPA approved and secure, there are no risks associated with its use. However, patients will be asked to ensure that they are in a private room during the group sessions, to protect their privacy and that of group members.
As with any group studies, there may be confidentiality issues, but we will discuss the importance of maintaining confidentiality at the beginning of each group.

VIII. POTENTIAL BENEFITS

Patients will be informed that there may be no direct benefit from participating in this research study. Some patients may become better able to cope with stress and NF symptoms, and experience improved quality of life.

In the future, knowledge from this research study may benefit others by providing information on how such interventions can help patients with neurofibromatosis 1, neurofibromatosis 2, and schwannomatosis

IX. MONITORING AND QUALITY ASSURANCE

Electronic information will be stored in REDCap (Research Electronic Data Capture), a free, secure, and HIPAA-compliant web-based application hosted by the Partners HealthCare Research Computing Enterprise Research Infrastructure & Services (ERIS) group (based at the PHS Needham corporate datacenter). Data will be stored on password protected computers that will be stored in secure locations at all times. Paper data files (with coded subject identification) will be stored in a locked filing cabinet. Only research staff will have access to these data locations.

A unique anonymous identifier will be assigned to each subject; subsequently, all data collected will be associated exclusively with this identifier. This includes all questionnaires administered over the course of the study, as well as home practice logs. Data from this study will be stored for three years after the publication of all study results, at which time all paper data files will be shredded and computer files will be deleted.

Data Management and Quality Control Procedures

To maximize accuracy and security, all survey data will be collected and stored on REDCap. Research staff will ensure that proper consent has been obtained before sending the REDCap survey to each participant.

REDCap (Research Electronic Data Capture) is a free, secure, HIPAA compliant web-based application hosted by the Partners HealthCare Research Computing Enterprise Research Infrastructure & Services (ERIS) group. Vanderbilt University, with collaboration from a consortium of academic and non-profit institutional partners, has developed this software toolset and workflow methodology for electronic collection and management of research and clinical study data. Data collection projects rely on a study-specific data dictionary defined by members of the research team with planning assistance from Harvard Catalyst, The Harvard Clinical and Translational Science Center EDC Support Staff. This iterative development and testing process results in a well-planned data collection strategy for individual studies. Using REDCap, the research team can also design web-based surveys and engage potential respondents using a variety of notification methods. REDCap provides flexible features that can be used for a variety of research projects and provides an intuitive interface to enter data with real time validation (automated data type and range checks). The system offers easy data manipulation with audit trails, reports for monitoring and querying participant records, and an automated export mechanism to common statistical packages (SPSS, SAS, Stata, R/S-Plus).

Since consistency of application of the study protocol is critical to acquiring high quality data, all research personnel have undergone or will undergo a competency-based training program prior to enrolling subjects.

Data and Safety Monitoring Plan

Adverse Event Monitoring: Throughout the study subjects will be monitored for the occurrence of events defined as any undesirable experience or unanticipated risk. Lack of effect of treatment is not considered an event. All adverse events will be reported on an adverse event form. The Principle Investigator has the responsibility of reporting serious adverse events (death, life threatening illness or injury, serious injury, or permanent disability) to PHRC within 24-72 hours of notification.
