# Supplementary material for: Effect of Mind-Body Skills Training on Quality of Life for Geographically Diverse Adults With Neurofibromatosis: A Fully Remote Randomized Clinical Trial
Source: JAMA Netw Open. 2023 Jun 28;6(6):e2320599. doi: 10.1001/jamanetworkopen.2023.20599 (PMC10308247; doi:10.1001/jamanetworkopen.2023.20599)
Supplement: Supplement 2. — eAppendix 1. Code, Equations, and Descriptions of the Linear Mixed Models eTable 1. Within- and Between-Group Differences in Quality of Life Domains From Baseline to Post-Treatment, Post 6-Months, and Post 12-Months eTable 2. Durability of Treatment Effects Measured by Within- and Between-Group Differences in Quality of Life Domains From Post-Treatment to Follow-up eAppendix 2. Projected Timeline for Patient Groups and Data Collection (Simplified) eReferences eAppendix 3. Details on The 3RP-NF Intervention [file jamanetwopen-e2320599-s002.pdf]

Supplementary Online Content

Vranceanu AM, Manglani HR, Choukas NR, et al. Effect of mind-body skills training on quality of life for geographically diverse adults with neurofibromatosis: a fully remote randomized clinical trial. *JAMA Netw Open.* 2023;6(6):e2320599. doi:10.1001/jamanetworkopen.2023.20599

eAppendix 1. Code, Equations, and Descriptions of the Linear Mixed Models
eTable 1. Within- and Between-Group Differences in Quality of Life Domains From Baseline to Post- Treatment, Post 6-Months, and Post 12-Months
eTable 2. Durability of Treatment Effects Measured by Within- and Between-Group Differences in Quality of Life Domains From Post-Treatment to Follow-up
eAppendix 2. Projected Timeline for Patient Groups and Data Collection (Simplified)
eReferences.
eAppendix 3. Details on The 3RP-NF Intervention

This supplementary material has been provided by the authors to give readers additional information about their work.

© 2023 Vranceanu AM et al. *JAMA Network Open.*

eAppendix. Code, Equations, and Descriptions of the Linear Mixed Models

Code:

```
 proc mixed data=import;
  by  vord vname vlabel ;
*  where vord = 1;
  class subject_id treatment block visit  ;
  model value = visit  post*treatment*visit   / solution cl;
  random visit / subject=block type=un;
  repeated visit / subject=subject_id(block) type=UN ;
  estimate "Active|intercept |Wk 00" intercept 1 visit  1  0  0  0 post*treatment*visit 0  0  0  0   0  0  0  0 / cl;
  estimate "Active|intercept |Wk 04" intercept 1 visit  0  1  0  0 post*treatment*visit 0  0  0  0   0  1  0  0 / cl;
  estimate "Active|intercept |Wk 24" intercept 1 visit  0  0  1  0 post*treatment*visit 0  0  0  0   0  0  1  0 / cl;
  estimate "Active|intercept |Wk 52" intercept 1 visit  0  0  0  1 post*treatment*visit 0  0  0  0   0  0  0  1 / cl;
  estimate "Active |within |Differences between weeks 0 to 4" intercept 0 visit -1  1  0  0 post*treatment*visit 0  0  0  0   0  1
0  0 / cl;
  estimate "Active |within |Differences between weeks 0 to 24" intercept 0 visit -1  0  1  0 post*treatment*visit 0  0  0  0   0  0
1  0 / cl;
  estimate "Active |within |Differences between weeks 0 to 52" intercept 0 visit -1  0  0  1 post*treatment*visit 0  0  0  0   0  0
0  1 / cl;
  estimate "Control | intercept |Wk 00" intercept 1 visit  1  0  0  0 post*treatment*visit 0  0  0  0   0  0  0  0 / cl;
  estimate "Control | intercept |Wk 04" intercept 1 visit  0  1  0  0 post*treatment*visit 0  1  0  0   0  0  0  0 / cl;
  estimate "Control |intercept |Wk 24" intercept 1 visit  0  0  1  0 post*treatment*visit 0  0  1  0   0  0  0  0 / cl;
  estimate "Control |intercept |Wk 52" intercept 1 visit  0  0  0  1 post*treatment*visit 0  0  0  1   0  0  0  0 / cl;
  estimate "Control |within |Differences between weeks 0 to 04" intercept 0 visit -1  1  0  0 post*treatment*visit 0  1  0  0   0
0  0  0 / cl;
  estimate "Control |within |Differences between weeks 0 to 24" intercept 0 visit -1  0  1  0 post*treatment*visit 0  0  1  0   0
0  0  0 / cl;
  estimate "Control |within |Differences between weeks 0 to 52" intercept 0 visit -1  0  0  1 post*treatment*visit 0  0  0  1   0
0  0  0 / cl;
  estimate "Active vs Control |between |Differences between weeks 0 to 04" intercept 0 visit  0  0  0  0 post*treatment*visit 0
-1  0  0   0  1  0  0 / cl;
  estimate "Active vs Control |between |Differences between weeks 0 to 24" intercept 0 visit  0  0  0  0 post*treatment*visit 0
0 -1  0   0  0  1  0 / cl;
  estimate "Active vs Control |between |Differences between weeks 0 to 52" intercept 0 visit  0  0  0  0 post*treatment*visit 0
0  0 -1   0  0  0  1 / cl;
  estimate "Active |within |Differences between weeks 4 to 24" intercept 0 visit  0 -1  1  0 post*treatment*visit 0  0  0  0   0 -1
1  0 / cl;
  estimate "Active |within |Differences between weeks 4 to 52" intercept 0 visit  0 -1  0  1 post*treatment*visit 0  0  0  0   0 -1
0  1 / cl;
  estimate "Control |within |Differences between weeks 4 to 24" intercept 0 visit  0 -1  1  0 post*treatment*visit 0 -1  1  0   0
0  0  0 / cl;
  estimate "Control |within |Differences between weeks 4 to 52" intercept 0 visit  0 -1  0  1 post*treatment*visit 0 -1  0  1   0
0  0  0 / cl;
  estimate "Active vs Control |between |Differences between weeks 4 to 24" intercept 0 visit  0  0  0  0 post*treatment*visit 0
1 -1  0   0 -1  1  0 / cl;
  estimate "Active vs Control |between |Differences between weeks 4 to 52" intercept 0 visit  0  0  0  0 post*treatment*visit 0
1  0 -1   0 -1  0  1 / cl;
  ods output  Estimates = work.est02   ;
run;
```

* vord, vname, and vlabel are used to define the 4 dimensions of QoL in the data set.

Equation:

© 2023 Vranceanu A-M et al. *JAMA Network Open.*

$value_{ij} = \beta_0 + \beta_1 * visit_{ij} + \beta_2 * post_{ij} * treatment_{ij} * visit_{ij} + u_{k(i)} + u_{i(k)} + e_{ij}$

"Active|intercept |Wk 00": $\beta_0*1 + \beta_1 * 1 + \beta_2 * 0 + \beta_3 * 0 + \beta_4 * 0 + \beta_5 * 0 + \beta_6 * 0 + \beta_7 * 0 + \beta_8 * 0+ \beta9 * 0 + \beta10 *0 + \beta11 * 0 + \beta12* 0$

"Active|intercept |Wk 04": $\beta_0*1 + \beta_1 * 0 + \beta_2 * 1 + \beta_3 * 0 + \beta_4 * 0 + \beta_5 * 0 + \beta_6 * 1+ \beta_7 * 0 + \beta_8 * 0+ \beta9 * 0 + \beta10 * 0 + \beta11 * 0 + \beta12* 0$

"Active|intercept |Wk 24": $\beta_0*1 + \beta_1 * 0 + \beta_2 * 0 + \beta_3 * 1 + \beta_4 * 0 + \beta_5 * 0 + \beta_6 * 0 + \beta_7 * 1 + \beta_8 * 0+ \beta9 * 0 + \beta10 * 0 + \beta11 * 0 + \beta12* 0$

"Active|intercept |Wk 52": $\beta_0*1 + \beta_1 * 0 + \beta_2 * 0 + \beta_3 * 0 + \beta_4 * 1 + \beta_5 * 0 + \beta_6 * 0 + \beta_7 * 0 + \beta_8 * 1 + \beta9 * 0 + \beta10 * 0 + \beta11 * 0 + \beta12* 0$

"Active |within |Differences between weeks 0 to 4": $\beta_0*0 + \beta_1 * (-1) + \beta_2 * 1 + \beta_3 * 0 + \beta_4 * 0 + \beta_5 * 0 + \beta_6 * 1 + \beta_7 * 0 + \beta_8 * 0+ \beta9 * 0 + \beta10 * 0 + \beta11 * 0 + \beta12* 0$

"Active |within |Differences between weeks 0 to 24": $\beta_0*0 + \beta_1 * (-1) + \beta_2 * 0 + \beta_3 * 1 + \beta_4 * 0 + \beta_5 * 0 + \beta_6 * 0 + \beta_7 * 1 + \beta_8 * 0+ \beta9 * 0 + \beta10 * 0 + \beta11 * 0 + \beta12* 0$

"Active |within |Differences between weeks 0 to 52": $\beta_0*0 + \beta_1 * (-1) + \beta_2 * 0 + \beta_3 * 0 + \beta_4 * 1 + \beta_5 * 0 + \beta_6 * 0 + \beta_7 * 0 + \beta_8 * 1+ \beta9 * 0 + \beta10 * 0 + \beta11 * 0 + \beta12* 0$

"Control | intercept |Wk 00": $\beta_0*1 + \beta_1 * 1 + \beta_2 * 0 + \beta_3 * 0 + \beta_4 * 0 + \beta_5 * 0 + \beta_6 * 0 + \beta_7 * 0 + \beta_8 * 0+ \beta9 * 0 + \beta10 * 0 + \beta11 * 0 + \beta12* 0$

"Control | intercept |Wk 04": $\beta_0*1 + \beta_1 * 0 + \beta_2 * 1 + \beta_3 * 0 + \beta_4 * 0 + \beta_5 * 0 + \beta_6 * 0 + \beta_7 * 0 + \beta_8 * 0+ \beta9 * 0 + \beta10 * 1 + \beta11 * 0 + \beta12* 0$

"Control |intercept |Wk 24": $\beta_0*1 + \beta_1 * 0 + \beta_2 * 0 + \beta_3 * 1 + \beta_4 * 0 + \beta_5 * 0 + \beta_6 * 0 + \beta_7 * 0 + \beta_8 * 0+ \beta9 * 0 + \beta10 * 0 + \beta11 * 1 + \beta12* 0$

"Control |intercept |Wk 52": $\beta_0*1 + \beta_1 * 0 + \beta_2 * 0 + \beta_3 * 0 + \beta_4 * 1 + \beta_5 * 0 + \beta_6 * 0 + \beta_7 * 0 + \beta_8 * 0+ \beta9 * 0 + \beta10 * 0 + \beta11 * 0 + \beta12* 1$

"Control |within |Differences between weeks 0 to 04": $\beta_0*0 + \beta_1 * (-1) + \beta_2 * 1 + \beta_3 * 0 + \beta_4 * 0 + \beta_5 * 0 + \beta_6 * 0 + \beta_7 * 0 + \beta_8 * 0+ \beta9 * 0 + \beta10 * 1 + \beta11 * 0 + \beta12* 0$

"Control |within |Differences between weeks 0 to 24": $\beta_0*0 + \beta_1 * (-1) + \beta_2 * 0 + \beta_3 * 1 + \beta_4 * 0 + \beta_5 * 0 + \beta_6 * 0 + \beta_7 * 0 + \beta_8 * 0+ \beta9 * 0 + \beta10 * 0 + \beta11 *1 + \beta12* 0$

"Control |within |Differences between weeks 0 to 52": $\beta_0*0 + \beta_1 * (-1) + \beta_2 * 0 + \beta_3 * 0 + \beta_4 * 1 + \beta_5 * 0 + \beta_6 * 0 + \beta_7 * 0 + \beta_8 * 0+ \beta9 * 0 + \beta10 * 0 + \beta11 * 0 + \beta12* 1$

"Active vs Control |between |Differences between weeks 0 to 04": $\beta_0*0 + \beta_1 * 0 + \beta_2 * 0 + \beta_3 * 0 + \beta_4 * 0 + \beta_5 * 0 + \beta_6 * 1 + \beta_7 * 0 + \beta_8 * 0+ \beta9 * 0 + \beta10 * (-1) + \beta11 * 0 + \beta12* 0$

"Active vs Control |between |Differences between weeks 0 to 24": $\beta_0*0 + \beta_1 * 0 + \beta_2 * 0 + \beta_3 * 0 + \beta_4 * 0 + \beta_5 * 0 + \beta_6 * 0 + \beta_7 * 1 + \beta_8 * 0+ \beta9 * 0 + \beta10 * 0 + \beta11 * (-1) + \beta12* 0$

"Active vs Control |between |Differences between weeks 0 to 52": $\beta_0*0 + \beta_1 * 0 + \beta_2 * 0 + \beta_3 * 0 + \beta_4 * 0 + \beta_5 * 0 + \beta_6 * 0 + \beta_7 * 0 + \beta_8 * 1+ \beta9 * 0 + \beta10 * 0 + \beta11 * 0 + \beta12* (-1)$

"Active |within |Differences between weeks 4 to 24": $\beta_0*0 + \beta_1 * 0 + \beta_2 * (-1) + \beta_3 * 1 + \beta_4 * 0 + \beta_5 * 0 + \beta_6 * (-1) + \beta_7 * 1 + \beta_8 * 0+ \beta9 * 0 + \beta10 * 0 + \beta11 * 0 + \beta12* 0$

"Active |within |Differences between weeks 4 to 52": $\beta_0*0 + \beta_1 * 0 + \beta_2 * (-1) + \beta_3 * 0 + \beta_4 * 1 + \beta_5 * 0 + \beta_6 * (-1) + \beta_7 * 0 + \beta_8 * 1 + \beta9 * 0 + \beta10 * 0 + \beta11 * 0 + \beta12* 0$

"Control |within |Differences between weeks 4 to 24": $\beta_0*0 + \beta_1 * 0 + \beta_2 * (-1) + \beta_3 * 1 + \beta_4 * 0 + \beta_5 * 0 + \beta_6 * 0 + \beta_7 * 0 + \beta_8 * 0+ \beta9 * 0 + \beta10 * (-1) + \beta11 * 1 + \beta12* 0$

"Control |within |Differences between weeks 4 to 52": $\beta_0*0 + \beta_1 * 0 + \beta_2 * (-1) + \beta_3 * 0 + \beta_4 * 1 + \beta_5 * 0 + \beta_6 * 0 + \beta_7 * 0 + \beta_8 * 0+ \beta9 * 0 + \beta10 * (-1) + \beta11 * 0 + \beta12* 1$

"Active vs Control |between |Differences between weeks 4 to 24": $\beta_0*0 + \beta_1 *0 + \beta_2 * 0 + \beta_3 * 0 + \beta_4 * 0 + \beta_5 * 0 + \beta_6 * 1 + \beta_7 * (-1) + \beta_8 * 0+ \beta9 * 0 + \beta10 * (-1) + \beta11 * 1 + \beta12* 0$

"Active vs Control |between |Differences between weeks 4 to 52": $\beta_0*0 + \beta_1 * 0 + \beta_2 * 0 + \beta_3 * 0 + \beta_4 * 0 + \beta_5 * 0 + \beta_6 * 1 + \beta_7 * 0 + \beta_8 * (-1) + \beta9 * 0 + \beta10 * (-1) + \beta11 * 0 + \beta12* 1$

Descriptive:

- $value_{ij}$ is the dependent variable for subject i at visit j
- $\beta0$ is the intercept of the fixed-effect
- β_1 and β_2 are fixed-effect coefficients for the main effect of visit and the 3-way interaction between post, treatment, and visit, respectively.
- $visit_{ij}$ is a categorical variable displaying the visit for subject i at time point j.
- $post_{ij} * treatment * visit_{ij}$ displays the 3-way interaction term.

© 2023 Vranceanu A-M et al. *JAMA Network Open.*

- post_ij is a dichotomous variable implying the post-baseline status (0=baseline, 1=post-baseline) for subject i at visit j
- treatment_ij is a dichotomous variable implying the treatment group (active or control) for subject i at visit j
- u_k(i) is a random intercept for block k, with k being the block that subject i is a member of (with u_k ~ N(0, σ_u^2)). Since the treatments were in group format, variable block was created to represent this level of grouping of individual participants.
- u_i(k) is a random effect for the visit variable within subject i, which is further nested within the block variable
- e_ij is the residual error term for subject i at time point j
- The ESTIMATE statements are linear combinations of the fixed-effects parameters that are used to conduct particular contrasts of interest. Each estimate statement begins with a label (e.g., " Active|intercept |Wk 00"), is followed by the specification of the linear contrasts involving the model parameters, and ends with the "/cl" option for producing confidence limits.

The first ESTIMATE statement, for example, is "Active|intercept |Wk 00" intercept 1 wave. 1 0 0 0 visit after treatment. This statement computes the estimate of the outcome at week 0 (baseline) for the active group. The linear combination is as follows: $\beta_0 * 1 + \beta_1 * 1 + \beta_2 * 0 + \beta_3 * 0 + \beta_4 * 0 + \beta_5 * 0 + \beta_6 * 0 + \beta_7 * 0 + \beta_8 * 0 + \beta 9 * 0 + \beta 10 * 0 + \beta 11 * 0 + \beta 12 * 0 = \beta\_0 + \beta\_1$
Each ESTIMATE statement that follows computes distinct contrasts or changes over time for individual groups or between groups.

© 2023 Vranceanu A-M et al. *JAMA Network Open.*

eTable 1. Within- and Between-Group Differences in Quality of Life Domains From Baseline to Post-Treatment, Post 6-Months, and Post 12-Months

	Baseline to Post-treatment			Baseline to 6-month post			Baseline to 12-month post		
	Mean Difference (95% CI)	*P-value*	*ES*	*Mean Difference (95% CI)*	*P-value*	*ES*	*Mean Difference (95% CI)*	*P-value*	*ES*
**Physical QoL**									
Within 3RP-NF	5.10; 3.21– 6.99	<.001	0.26	6.51; 4.15 – 8.87	<.001	0.33	6.33; 4.13 – 8.52	<.001	0.32
Within HEP-NF	6.42; 4.55 – 8.29	<.001	0.31	4.66; 2.35 – 6.96	<.001	0.22	2.75; .618– 4.89	.01	0.13
Between	-1.32; -3.88 − 1.23	.31	-0.07	1.85; -1.37 – 5.08	.26	0.09	3.57; .517– 6.57	.02	0.18
**Psychological QoL**									
Within 3RP-NF	8.54; 6.42 – 10.7	<.001	0.49	7.98; 5.73 – 10.2	<.001	0.46	8.43; 5.32 – 11.5	<.001	0.49
Within HEP-NF	9.15; 7.06 – 11.2	<.001	0.52	6.82; 4.68 – 8.97	<.001	0.39	5.31; 2.26 – 8.36	.001	0.30
Between	-0.60; -3.34− 2.14	.67	-0.03	1.15; 1.85 – 4.15	.45	0.07	3.11; -1.01 – 7.24	.14	0.18
**Social QoL**									
Within 3RP-NF	6.67; 3.11 – 10.2	.001	0.29	6.81; 3.09 – 10.5	.001	0.30	9.68; 5.29 – 14.1	<.001	0.43
Within HEP-NF	5.83; 2.32 – 9.34	.001	0.26	5.46; 1.88– 9.05	.003	0.24	2.75; -1.55 – 7.07	.21	0.12
Between	0.83; -4.06− 5.73	.74	0.04	1.34; -3.58 – 6.28	.59	0.06	6.92; 1.17 – 12.7	.02	0.31
**Environmental QoL**									
Within 3RP-NF	3.74; 1.59 – 5.89	.001	0.23	4.85; 2.63 – 7.07	<.001	0.29	7.28; 4.99 – 9.56	<.001	0.44
Within HEP-NF	5.48; 3.33 – 7.62	<.001	0.33	4.27; 2.11 – 6.44	.001	0.26	3.79; 1.56 – 6.02	.001	0.23
Between	-1.73; -4.60 – 1.14	0.24	-0.11	0.57; -2.42 – 3.57	.71	0.03	3.48; .430– 6.54	.02	0.21

***Note*: QoL = Quality of Life; 3RP-NF = Relaxation Response Resiliency Program for Neurofibromatosis; HEP-NF = Health Enhancement Program for NF. ES = effect size (standardized estimates).**

© 2023 Vranceanu A-M et al. *JAMA Network*

eTable 2. Durability of Treatment Effects Measured by Within- and Between-Group Differences in Quality of Life Domains From Post-Treatment to Follow-up

	**Post-treatment to 6-month post**				**Post-treatment to 12-month post**			
	Mean Difference (95% CI)	*P-value*	*ES*	*lower one-sided 95% confidence bound*	*Mean Difference (95% CI)*	*P-value*	*ES*	*lower one-sided 95% confidence bound*
**Physical QoL**								
Within 3RP-NF	1.46; -0.77 - 3.60	.20	0.075		1.23; -.810 - 3.27	.24	0.06	
Within HEP-NF	-1.76; -3.87 -.347	.10	-0.09		-3.74; -5.62 - -1.70	.001	-0.18	
Between	3.18; .140- 6.22	.04	0.16	.630	4.89; 2.07 - 7.73	.001	0.25	2.52
**Psychological QoL**								
Within 3RP-NF	-.560; -3.16 - 2.03	.67	-0.03		-.117; -2.94 - 2.70	.93	-0.01	
Within HEP-NF	-2.32; -4.84 - .198	.07	-0.13		-3.83; -6.57 - 1.09	.006	-0.22	
Between	1.75; -1.84 - 5.36	.34	0.10	-1.26	3.71; - .211 - 7.64	.06	0.21	.422
**Social QoL**								
Within 3RP-NF	1.14; -2.71 - 3.00	.92	0.05		3.01; -0.35 - 6.39	.08	0.13	
Within HEP-NF	-.363; -3.07- 2.34	.79	-0.02		-3.07; -6.34- .193	.06	-0.14	
Between	.510; -3.38 - 4.40	.79	0.02	-2.75	6.09; 1.56 - 10.6	.008	0.27	2.29
**Environmental QoL**								
Within 3RP-NF	1.10; -0.65 - 2.86	.22	0.06		3.53; 1.30 - 5.75	.001	0.22	
Within HEP-NF	-1.20; -2.87 - .46	.16	-0.07		-1.68; -3.84- .471	.12	-0.10	
Between	2.31; -.110 - 4.72	.06	0.14	.279	5.21; 2.12 - 8.31	.001	0.32	2.62

***Note*: QoL = Quality of Life; 3RP-NF = Relaxation Response Resiliency Program for Neurofibromatosis; HEP-NF = Health Enhancement Program for NF. ES = effect size (standardized estimates).**

© 2023 Vranceanu A-M et al. *JAMA Network*

eAppendix 2. Projected Timeline for Patient Groups and Data Collection (Simplified)

Year 1	Year 2	Year 3	Year 4
Develop study protocol and train study staff (2 months)<br> Treat N=40 participants<br>(2 groups: one intervention and one control with 5-8 participants each within 2 months; 9 months)<br>* Start collecting 6 month follow up data	* Treat N = 92 participants (4 groups of 5-8 participants, 8 sessions each within 2 months; 12 months)<br>* Collect 6 month follow up data<br>* Start collecting 12 month follow up data	* Treat N=92 participants<br>* (4 groups of 5-8 participants, 8 sessions each within 2 months; 12 months)<br>* Finalize 6 month follow up data<br>* Collect 12 month follow up data	* Finalize 12 month follow up data collection<br>* Data analyses<br>* Dissemination of results (conferences and manuscripts)<br>* Finalize plan for implementation of intervention within NF practices (NF Annual Forum, NF symposiums in the U.S., CTF Annual meeting trainings)<br>* Finalize implementation of intervention within NF practices

© 2023 Vranceanu A-M et al. *JAMA Network Open.*

eReferences.

1. Vranceanu AM, Merker VL, Park E, Plotkin SR. Quality of life among adult patients with neurofibromatosis 1, neurofibromatosis 2 and schwannomatosis: a systematic review of the literature. J Neurooncol. 2013 Sep; 114(3): 257-62. DOI:10.1007/s11060-013-1195-2.
2. Vranceanu AM, Riklin E, Merker V, Plotkin SR, Park ER. Psychosocial presentation of adults with Neurofibromatosis 1, 2 and schwannomatosis enrolled into a stress reduction program. Children's Tumor Foundation Annual Meeting, Monterey CA June 6-9, 2015.
3. Wang DL, Smith KB, Esparza S, Leigh FA, Muzikansky A, Park ER, Plotkin SR. Emotional functioning of patients with neurofibromatosis tumor suppressor syndrome. Genet Med. 2012 Dec; 14(12): 977-82.
4. Rumsey N, Harcourt D. Body image and disfigurement: issues and interventions. Body Image. 2014 Jan; 1(1): 83-97.
5. Brantley PJ, Dutton GR, Grothe KB, Bodenlos JS, Howe J, Jones GN. Minor life events as predictors of medical utilization in low income African American family practice patients. J Behav Med. 2005 Aug; 28(4): 395-401.
6. Gortmaker SL, Eckenrode J, Gore S. Stress and the utilization of health services: a time series and cross-sectional analysis. Journal of Health and Social Behavior. 1982 Mar; 23: 25-38.
7. Borell-Carrio F, Suchman AL, Epstein RM. The biopyschosocial model 25 years later: principles, practice, and scientific inquiry. Annals of Family Medicine. 2004; 2(6).
8. Carlson LE, Bultz BD. Efficacy and medical cost offset of psychosocial interventions in cancer care: making the case for economic analyses. Psycho-Oncology. 2004; 13: 837–849.
9. Vranceanu AM, Riklin E, Merker V, Macklin E, Park ER, Plotkin SR. Mind body therapy via videoconferencing in patients with NF. An RCT. Neurology 2016; in press.
10. Vranceanu AM, Merker VL, Plotkin SR, Park ER. The Relaxation Response Resilency Program (3RP) in patients with neurofibromatosis 1, neurofibromatosis 2, and schwannomatosis: results from a pilot study. J Neurooncol. 2014; 120(1): 1003-9.
11. Hommel KA, Hente E, Denson LA. Telehealth behavioral treatment for medication nonadherence: a pilot and feasibility study. European Journal of Gastroenterology & Hepatology. 2013 Apr; 25(4): 469-473.
12. Yuen EK, Herbert JD, Forman EM, Goetter EM, Juarascio AS, Rabin S, Goodwin C, Bouchard S. Acceptance based behavior therapy for social anxiety disorder through videoconferencing. J Anxiety Disord. 2013 May; 27(4): 389-97.
13. Williams A, LaRocca R, Chang T, Trinh NH, Fava M, Kvedar J, Yeung A. Web-based depression screening and psychiatric consultation for college students: a feasibility and acceptability study. Internation Journal of Telemedicine and Applications. 2014; 2014.
14. Wantanabe SM, Faichild A, Pituskin E, Borgersen P, Hanson J, Fassbender K. Improving access to specialist multidisciplinary palliative care consultation for rural cancer patients by videoconferencing: report of a pilot project. Support Care Center. 2013 Apr; 21(4): 1201-7.
15. Carlson LE, Lounsberry JJ, Maciejewski O, Wright K, Collacutt V, Taenzer P. Telehealth-delivered group smoking cessation for rural and urban participants: feasibility and cessation rates. Addictive Behaviors. 2012 Jan; 37(1): 108-14.
16. Park ER, Traeger L, Vranceanu AM, Scult M, Lerner JA, Benson H, Denninger J, Fricchione GL. The development of a patient-centered program based on the relaxation response: the Relaxation Response Resiliency Program (3RP). Psychosomatics. 2013 Mar-Apr; 54(2): 165-74. DOI:10.1016/j.psym.2012.09.001.
17. Benson H, Rosner BA, Marzetta BR, Klemchuk HM. Decreased blood pressure in pharmacologically treated hypertensive patients who regularly elicited the relaxation response. Lancet. 1974; i: 289-91.
18. Benson H, Klemchuk HP, Graham JR. The usefulness of the relaxation response in the therapy of headache. Headache. 1974; 14: 49-52.
19. Beary JF, Benson H. A simple psychophysiologic technique which elicits the hypometabolic changes of the relaxation response. Psychosomatic Med. 1974; 36: 115-20.
20. Benson H, Rosner BA, Marzetta BR, Klemchuk H. Decreased blood pressure in borderline hypertensive subjects who practiced meditation. J Chronic Dis. 1974; 27: 163-9.
21. Peters RK, Benson H, Porter D. Daily relaxation response breaks in a working population. Am J Public Health. 1977; 67: 946-59.

22. Greenwood MM, Benson H. The efficacy of progressive relaxation in systematic desensitization and a proposal for an alternative competitive response - the relaxation response. Behav Res Ther (Behaviour research and therapy). 1977; 15: 337-43.
23. Benson HB, Klipper MZ. The Relaxation Response. New York, NY: HarperTorch, 1975.
24. Dusek J, Otu HH, Wohlhueter AL, Bhasin M, Zerbini LF, Joseph MG, Benson H, Libermann TA. Genomic counter-stress changes induced by the relaxation response. 2008 July; 3(7): e2576. DOI: 10.1371/journal.pone.0002576.
25. Vranceanu AM, Shaefer JR, Saadi AF, Slawsby E, Sarin J, Scult M, Benson H, Denninger JW. The Relaxation Response Resiliency Enhancement Program in the management of Chronic Refractory Temporomandibular Joint Disorder: results from a pilot study. Journal of Musculoskeletal Pain. 2013; 3(21): 224-230. DOI:10.3109/10582452.2013.827289.
26. Bhasin MK, Dusek JA, Chang BH, Denninger JW, Fricchione GL, Benson H, Libermann TA. Relaxation response induces temporal transcriptome changes in energy metabolism, insulin secretion and inflammatory pathways. PLoS One. 2013 May; 8(5): e62817
27. Vranceanu AM, Gonzalez A, Niles H, Fricchione G, Baim M, Young A, Denninger JW, Park ER. Exploring the effectiveness of a modified comprehensive mind-body intervention for medical and psychological symptom relief. Psychosomatics. 2014; DOI:10.1016/j.psym.2014.01.005.
28. Denninger J, Jacquart J, Miller K, Radossi A, Haime V, Macklin E, Gilburd D, Oliver MN, Mehta D, Yeung A, Fricchione G, Benson H. The effectiveness of a community-based mind body group intervention for depression & anxiety: a pilot study. J Altern Complement Med. 2014 May; 20(5): A55. DOI:10.1089/acm.2014.5142.abstract.
29. Mehta D, Chittenden E, Denninger J, Haime V, Traeger L, Jackson V, Park E. Promoting resiliency among palliative care clinicians: a pilot intervention. J Altern Complement Med. 2014 May; 20(5): A102. DOI:10.1089/acm.2014.5270.abstract.
30. Denninger JW, Bhasin M, Huffman J, Niles H, Creager M, Pande R, Liberman T, Fricchione G, Benson H, Zusman R. Clinical and genomic effects of a relaxation response-based mind-body intervention in stage I hypertension. J Altern Complement Med. 2014 May; 20(5): A54. DOI:10.1089/acm.2014.5141.abstract.
31. Kuo B, Bhasin M, Jacquart J, Scult MA, Slipp L, Riklin E, Lepoutre V, Comosa N, Norton BA, Dassatti A, Rosenblum J, Thurler AH, Surjanhata BC, Hasheminejad NH, Kagan L, Slawsby E, Rao SR, Macklin EA, Fricchione GL, Benson H, Libermann TA, Korzenik, Denninger JW. Genomic and Clinical Effects Associated with a Relaxation Response Mind-Body Intervention in Patients with Irritable Bowel Syndrome and Inflammatory Bowel Disease. PLoS ONE 2015 April; DOI:10.1371/journal.pone.0123861
32. Miller KM, Chad-Friedman E, Haime V, Mehta DH, Lepoutre V, Gilburd D, Peltier-Saxe D, Lilley C, Benson H, Fricchione GL, Denninger JW, Yeung A. The effectiveness of a brief mind-body intervention for treating Depression in community health center patients. *Global Adv Health Med.* 2015 March; 4(2): 30-35.
33. Den Oudsten BL, Zijlstra WP, De Vries J. The minimal clinical important difference in the World Health Organization Quality of Life instrument--100. Support Care Cancer. 2013 May;21(5):1295–301.
34. Spitzer RL, Williams JBW, Kroenke K, Hornyak R, McMurray J. Validity and utility of the PRIME-MD Patient Health Questionnaire in assessment of 3000 obstetric-gynecologic patients: the PRIME-MD Patient Health Questionnaire Obstetrics-Gynecology Study. American Journal of Obstetrics and Gynecology. 2000; 183(3): 759-769.
35. Harris PA, Taylor R, Thielke R, Payne J, Gonzalez N, Conde JG. Research electronic data capture (REDCap) - A metadata-driven methodology and workflow process for providing translational research informatics support. J. of Biomedical Informatics. 2009 April; 42(2): 377-381. DOI: 10.1016/j.jbi.2008.08.010
36. Skevington SM, Lofty M, O'Connell KA. The World Health Organization's WHOQOL-BREF Quality of Life Assessment: Psychometric properties and results of the international field trial. A Report from the WHOQOL Group. *Quality of Life Research* 2004; 13: 299-310.
37. Wolters PL, Martin S, Merker VL, Gardner KL, Hingtgen CM, Tonsgard JH, Schorry EK, Baldwin A. Patient-reported outcomes in neurofibromatosis and schwannomatosis clinical trials. The Official Journal of the American Academy of Neurology. 2013 November 19; 81(21): S6-S14.
38. Spitzer RL, Kroenke K, Williams JB, Löwe B. A brief measure for assessing generalized anxiety disorder: the GAD-7. Arch Intern Med. 2006 May 22; 166(10): 1092-7.
39. Wilson D, Parson J, Tucker G. The SF-36 summary scales: problems and solutions. *Soc Prev Med*. 2000; 45: 239-246.

© 2023 Vranceanu A-M et al. *JAMA Network Open.*

40. Davis LL, Broome ME, Cox RP. Maximizing retention in community-based clinical trials. *J Nurs Scholarsh.* 2002;34(1):47–53.
41. Carroll KM, Rounsaville BJ. Bridging the gap: a hybrid model to link efficacy and effectiveness research in substance abuse treatment. *Psychiatr Serv.* 2003 Mar;54(3):333–9.
42. Liang KY, Zeger S. Longitudinal data analysis of continuous and discrete responses for pre–post designs. Sankhyā: The Indian Journal of Statistics (Series B) 2000; 62(1):134–148
43. Kleinbaum DG, Lawrence LK, Nizam A, Muller KE. Applied Regression Analysis and Other Multivariable Methods. Boston: Cengage Learning, 2007.
44. Lydick E, Epstein RS. Interpretation of quality of life changes. Qual Life Res. 1993; 2(3): 221-6.
45. MacKinnon DP, Lockwood CM, Hoffman JM, West SG, Sheets V. A comparison of methods to test mediation and other intervening variable effects. Psychol Methods. 2002 Mar; 7(1): 83.
46. Meeker WQ, Jr, Cornwell LW, Aroian LA. The product of two normally distributed random variables. In: Kennedy WJ, Odeh RE, editors. Selected tables in mathematical statistics. VII. Providence, RI: American Mathematical Society; 1981.
47. Ditlevsen S, Christensen U, Lynch J, Damsgaard MT, Keiding N. The mediation proportion: a structural equation approach for estimating the proportion of exposure effect on outcome explained by an intermediate variable. Epidemiology. 2005 Jan; 16(1): 114-20.
48. Baron RM, Kenny DA. The moderator-mediator variable distinction in social psychological research: conceptual, strategic, and statistical considerations. J Pers Soc Psychol. 1986 Dec; 51(6) 1173-82.
49. Kraemer HC, Wilson GT, Fairburn CG, Agras WS. Mediators and moderators of treatment effects in randomized clinical trials. Arch Gen Psychiatry. 2002 Oct; 59(10): 877-83.
50. Rounsaville BJ, Carroll KM, Onken LS. A stage model of behavioral therapies research: Getting started and moving from Stage 1. Clinical Psychology: Science and Practice. 2001; 8: 133-142.

© 2023 Vranceanu A-M et al. *JAMA Network Open.*

eAppendix 3. Details on The 3RP-NF Intervention

Description of the Intervention Relaxation Response Resiliency for NF:

Session 1: Stress/NF Symptom Management and Resiliency Training

Overview

In this session we start to look at how stress/NF symptoms affect each person, what are individual stressors/NF symptoms, and what resources each person has to cope with stress/NF symptoms. We start the session with an overview of the stress response and the relaxation response, and the concept of resiliency. We discuss how these apply to persons with NF. We examine the overall process involved in building resiliency and its three essential elements.

Session Content

Introduction to Mind Body Medicine

- The stress response in patients with NF
- The relaxation response in patients with NF
- Resiliency in patients with NF

Components of the 3RP-NF program

- Eliciting the relaxation response (RR)
- Stress/NF symptoms awareness
- Adaptive strategies

Guiding the elicitation of the RR

The weekly Practice Note – homework adherence

In-Session Exercises

Body awareness

RR Elicitation: Single-pointed focus meditation

RR Elicitation: Breath awareness

Energy battery

Between-Session Practice

Recorded skills given to each participant.

Complete the Practice Note

© 2023 Vranceanu A-M et al. *JAMA Network Open.*

- RR practice: single-pointed focus
- Daily appreciations
- Lifestyle behavior and social connectedness goals
- Symptoms check-in

Session 2: The Relaxation Response

Overview

In this session we explore different ways to elicit the RR and introduce the concept of “mini” RR-elicitation exercises. We practice the body awareness, and problem-solve common problems in eliciting the RR. We further explore the tendency to focus on negative emotions and physical sensations, and how these apply to NF patients. We discuss how diminished sleep can be made worse by or promote stress/increased NF symptoms and, conversely, how recuperative sleep can promote resiliency.

Session Content

Review of previous week’s between-session practice
- Weekly Practice Note
- Tips for developing a consistent practice

A closer look at the RR
- Overview of methods to elicit the RR
- Guidelines for eliciting the RR

The Mini

Recuperative sleep
- Sleep Tips
- The Sleep Diary

In-Session Exercises

RR Elicitation: Body Scan/Breath Focus

RR: Minis

Assessing Your Sleep

Identifying Emotions and Positive Physical Sensations general and NF specific

Between-Session Practice

Sleep diary

© 2023 Vranceanu A-M et al. *JAMA Network Open.*

Practice Note

Session 3: Stress Awareness in patients with NF

Overview

In this session, we explore how to decrease reactivity (impulsive quick reactions) to stress and medical symptoms, and we introduce the concept of mindfulness. We also develop awareness of the connection between stress, NF symptoms, thoughts, emotions, behaviors, and physical experiences and introduce the concept of social support. We discuss examples specific for patients with NF, such as meeting new people, communicating with doctors, appearance concerns, dating, and uncertainty of disease. We discuss the different types of social support, and we examine which types of social support you have, give, and utilize.

Session Content

Review

- Weekly Practice Note
- Sleep Diary

Mindful Awareness

Components of the Stress Response – specific examples for NF related stressors and general stressors

- Physical
- Cognitive
- Emotional
- Behavioral
- Relational

Social Support

In-Session Exercises

RR Elicitation: Mindful Awareness Meditations
Mindful Eating
Stress Warning Signals
The Social Support Diagram
End-of-Session Mini: Mindful Body Awareness

Between-Session Practice

Weekly Practice Note
New and Good
Mindful Awareness in Daily Living
Mindful Awareness Meditation

© 2023 Vranceanu A-M et al. *JAMA Network Open.*

<u>Session 4: Mending the NF patient's mind and body</u>

Overview
In this session we examine how stress, NF symptoms and our reactions to these affect both the mind and the body. We will discuss specifically how your individual NF stressors affect your mind and body. We begin this session by introducing a body-based RR technique: yoga. Then, we introduce the concepts of negative automatic thoughts and thought distortions and focus on adaptive strategies to cope with stress. We discuss specific example for each participant, and work together to help generate examples.

Session Content

Review

- Weekly Practice Note
- New and Good
- Mindful Awareness in Daily Living

Awareness of Movement

- Awareness of Movement in Daily Living

Negative Automatic Thoughts (general and NF specific)
Thought Distortions

In-Session Exercises

RR Elicitation: Yoga

End-of-Session Mini: Walking Meditation

Between-Session Practice

Weekly Practice Note

New and Good

Coping Log, Part 1

List of Pleasant Behaviors

<u>Session 5: Creating and adaptive perspective</u>

Overview
In this session we introduce guided imagery (learning to use imagination to feel better and learn about yourself). We continue to discuss the negative thoughts triggered by stress or NF symptoms

© 2023 Vranceanu A-M et al. *JAMA Network Open.*

and focus on how to change negative perspectives into adaptive ones. We deal with specific stressors encountered by patients with NF. We work on examples for all participants and help each other learn this skill through practice. We encourage ongoing reflection in order to create meaning from daily events that would otherwise have gone unnoticed. We discuss the value of purposeful engagement in pleasant behaviors and provide tips to remember to eat mindfully.

Session Content

Review

- Weekly Practice Note
- New and Good
- Coping Log, Part 1
- List of Pleasant Behaviors

Guided Imagery

Coping Log, Part 2

Healthy Eating

In-Session Exercises

RR Elicitation: Imagery

Creating Adaptive Perspectives

End-of-Session Mini: Joyful Place Imagery

Between-Session Practice

Weekly Practice Note

The Coping Log

Stop, Breathe, Reflect, Choose

Good, Bad, and Routine

<u>Session 6: Promoting Positivity</u>

Overview

We begin with a meditation that embodies the cultivation of positive, adaptive qualities such as kindness and love. Next, we expand our discussion of adaptive perspectives by looking at how we explain ourselves optimistically and pessimistically. We discussed specific examples on NF

© 2023 Vranceanu A-M et al. *JAMA Network Open.*

related situations. We look at how these can influence resiliency, stress, and NF symptoms respectively, and introduce methods of understanding the underlying fears that drive pessimistic thinking. We discuss pessimism in the context of NF and how to move toward optimism. Regular RR elicitation should be established by now, and we reflect on the experience of being in the RR, and contrast that to the experience of being in the stress response.

Session Content

Review

- Weekly Practice Note
- Good, Bad, and Routine
- The Coping Log
- List of Pleasant Behaviors

How We Tell Our Stories (Explanatory Style): Optimism vs. Pessimism

In-Session Exercises

RR Elicitation: Loving Kindness Meditation

Comparing Optimism and Pessimism

Relaxation Signals

End-of-Session Mini: I am… at peace

Between-Session Practice

Weekly Practice Note

The Coping Log

From Pessimism to Optimism

Root Fear

Session 7: Healing States of Mind

Overview

In this session we explore the practice of problem solving and acceptance as adaptive responses to stressful situations and NF symptoms. We deal specifically with NF stressors and symptoms that need to be accepted and some that can be problem solved. We practice a contemplation meditation

© 2023 Vranceanu A-M et al. *JAMA Network Open.*

and discuss the concept of empathy or perspective taking (e.g., putting yourself into the other person's shoes to really understand their perspective).

Session Content

Review
- Weekly Practice Note
- The Coping Log
- From Pessimism to Optimism
- Root fear

Coping Strategy: Problem-Solving vs. Acceptance

Empathy

In-Session Exercises

RR Elicitation: Contemplation

Achieving Acceptance

Mindful Awareness of Another

End-of-Session Mini: Contemplation

Between-Session Practice

Weekly Practice Note

Session 8: Humor, Empathy and Staying Resilient

Overview
In this session, we learn how to use humor and imagination to improve coping skills. We look in depth at the role of empathy for self and others in building and maintaining resiliency. We discussed empathy when it comes to people who may not understand or know about NF. We role-play ways to interact and explain NF symptoms. We also discuss the use of humor as a way of moving from SR to RR. We review the program content and plan for how to continue practicing after the program ends.

Session Content

Review
- Weekly Practice Note

- Letter to Self

Humor and Coping
- Laughter
- More Humor Strategies

Empathy

Staying Resilient
- Tips for Staying Resilient

In-Session Exercises

Laughter

Create a Sitcom from Your Life

Mindful Awareness of Another

Program Review

Group and individual

Review of RR practice note

© 2023 Vranceanu A-M et al. *JAMA Network Open.*
