# Supplementary material for: Effect of Mind-Body Skills Training on Quality of Life for Geographically Diverse Adults With Neurofibromatosis: A Fully Remote Randomized Clinical Trial
Source: JAMA Netw Open. 2023 Jun 28;6(6):e2320599. doi: 10.1001/jamanetworkopen.2023.20599 (PMC10308247; doi:10.1001/jamanetworkopen.2023.20599)
Supplement: Supplement 3. — Data Sharing Statement [file jamanetwopen-e2320599-s003.pdf]

Data Sharing Statement

Vranceanu. Effect of Mind-Body Skills Training on Quality of Life for Geographically Diverse Adults With Neurofibromatosis. *JAMA Netw Open*. Published June 28, 2023. doi:10.1001/jamanetworkopen.2023.20599

Data
Data available: Yes
Data types: Deidentified participant data
How to access data: Data will be made available for sharing upon request (avranceanu@mgh.harvard.edu).
When available: beginning date: 05-01-2025

Supporting Documents
Document types: Statistical/analytic code
How to access documents: The statistical/analytic code is available in the Supplemental Materials.
When available: With publication

Additional Information
Who can access the data: Data will be made available to researchers whose proposed use of the data has been approved.
Types of analyses: Data will be made available for a specified purpose.
Mechanisms of data availability: Data will be made available with a signed data access agreement.
